# Potential Antioxidant Compounds from the Spores of *Dicranopteris linearis* and the Branches of *Averrhoa bilimbi*

**DOI:** 10.3390/antiox13111319

**Published:** 2024-10-29

**Authors:** Thuc-Huy Duong, Thi-Minh-Dinh Tran, Phuong-Mai To, Nguyen-Hong-Nhi Phan, Thi-Phuong Nguyen, Huong Thuy Le, Jirapast Sichaem

**Affiliations:** 1 Department of Chemistry, Ho Chi Minh City University of Education, 280 An Duong Vuong Street, District 5, Ho Chi Minh City 700000, Vietnam; huydt@hcmue.edu.vn (T.-H.D.); tophuongmai0407@gmail.com (P.-M.T.); 2 Department of Biology, Ho Chi Minh City University of Education, 280 An Duong Vuong Street, District 5, Ho Chi Minh City 700000, Vietnam; dinhttm@hcmue.edu.vn; 3 NTT Hi-Tech Institute, Nguyen Tat Thanh University, 300A Nguyen Tat Thanh, District 4, Ho Chi Minh City 700000, Vietnam; pnhnhi@ntt.edu.vn (N.-H.-N.P.); nguyenphuong@ntt.edu.vn (T.-P.N.); 4 Faculty of Pharmacy, Ton Duc Thang University, Ho Chi Minh City 700000, Vietnam; 5 Research Unit in Natural Products Chemistry and Bioactivities, Faculty of Science and Technology, Thammasat University Lampang Campus, Lampang 52190, Thailand

**Keywords:** *Dicranopteris linearis*, *Averrhoa bilimbi*, bio-guided isolation, antioxidant activity, phenolic content, flavonoid content

## Abstract

This study focused on bio-guided isolation based on antioxidant activities from *Dicranopteris linearis* spores and *Averrhoa bilimbi* branches. The total phenolic content (TPC), total flavonoid content (TFC), and antioxidant activities of the extracts were determined. For *D. linearis* spores, the ethyl acetate (EA) extract exhibited the highest TPC (120.13 ± 0.04 mg GAE/g) and TFC (21.94 ± 0.30 mg QE/g), along with strong DPPH antioxidant activity (96.3 ± 0.3% inhibition, IC_50_ of 39.4 ± 0.3 µg/mL). For *A. bilimbi* branches, the *n*-hexane–ethyl acetate (HEA) extract showed the highest TPC (165.21 ± 0.24 mg GAE/g) and TFC (26.20 ± 0.01 mg QE/g), with significant DPPH antioxidant activity (89.6 ± 0.7% inhibition, IC_50_ of 39.7 ± 1.9 µg/mL). Phytochemical investigation led to the identification of ten compounds (**D1**–**D10**) from *D. linearis* spores and twelve compounds (**A1**–**A12**) from *A. bilimbi* branches. Notably, compound **A1** was identified as a new natural compound. The chemical structures were elucidated through NMR spectroscopy and comparison with existing literature. The antioxidant activities of selected compounds (**D3**–**D5**, **D8**–**D10**, and **A1**–**A11**) were evaluated using DPPH and ABTS free radical scavenging assays. Among them, compound **A3** exhibited the strongest antioxidant activities (IC_50_ of 7.1 ± 0.1 µg/mL for DPPH and 14.8 ± 0.1 for ABTS, respectively). The results of this study highlight the potential of *D. linearis* and *A. bilimbi* for use in natural product-based antioxidant applications.

## 1. Introduction

Comprehensive reviews have indicated that ferns are well known for their traditional uses, including hepatoprotective, antihyperglycemic, leishmanicidal, and trypanocidal activities [1,2]. *Dicranopteris linearis* (Burm. F.) Underw., a common fern widely distributed in Vietnam, is traditionally used in East Asian countries to treat various ailments such as fever (in Malaysia) and intestinal worms (in Indochina) [3]. In India, it is used for treating asthma and infertility in women, and in Papua New Guinea, it is used for wound healing [4]. Various pharmacological properties of *D. linearis* have been reported, including anticancer, antibacterial, antioxidant, analgesic, and anti-HIV activities [4,5,6]. The chemical composition of *D. linearis* has been thoroughly examined, with more than 40 compounds identified, predominantly in the leaves [4,5,6,7,8]. Several studies have also explored the pharmaceutical properties of *D. linearis* leaf extracts. For instance, Ponnusamy and colleagues studied the wound healing properties of a polar fraction of *D. linearis* leaves using a combination of in vitro assays, including DPPH, FRAP, and MTT [4]. The hepatoprotective potential of *D. linearis* extracts has also been demonstrated using different in vivo models, including CCl_4_-induced and paracetamol-induced rats [3,7,8]. More recently, the extract of *D. linearis* leaves was found to be a potent antidepressant agent in an in vivo study [9]. Our previous report indicated that *D. linearis* leaves might be a potent source of alpha-glucosidase inhibitors [10]. The antioxidant activity of Malaysian *D. linearis* leaves has been comprehensively studied using the DPPH method [4]. Zakaria and co-workers further demonstrated that both water-soluble and lipid-soluble extracts exhibited potent antioxidant and hepatoprotective activities [6,7,8,11,12].

*Averrhoa bilimbi* L. is an edible plant with numerous traditional uses. In India, the fruit is used as an antidiabetic agent [13] and for treating obesity [14,15]. It is also employed to treat whooping cough, pimples, and hypertension [15]. The leaves of the plant are used to treat fever and skin infections, and as an antiscorbutic and astringent [14,15]. A decoction prepared from the fruits is used to treat hepatitis, fever, and diarrhea [14]. However, little is known about the chemical constituents of *A. bilimbi*. The isolation of 14 components, including two new flavonoid glycosides, was previously reported from the leaves of *A. bilimbi* in Indonesia [16], showing potent inhibition towards cytochrome P450 3A4 and 2D6. Two other studies on the fruits of *A. bilimbi* in India reported HPLC screening data and the isolation of dihydromyricetin [17,18]. Despite the scarcity of chemical data, a significant number of biological investigations have been conducted on this plant. These include studies on its antioxidant [19,20], antiradical, xanthine oxidase inhibition [21], antibacterial [22,23,24], cytotoxic [14], and thrombolytic activities [24]. In vivo studies have also been conducted on its antidiabetic effect [13] and anti-ulcerative colitis activity [25].

Despite the considerable medicinal value demonstrated by *D. linearis* leaves and *A. bilimbi* fruits, the reproductive spores of *D. linearis* and the branches of *A. bilimbi* have not yet been thoroughly studied. Both parts of the plants may contain bioactive compounds that could contribute to the development of new therapeutic agents, particularly in the area of antioxidant research, which is crucial for managing oxidative stress-related diseases such as cancer, diabetes, and cardiovascular conditions. The goal of this study is to investigate the chemical composition and antioxidant activities of *D. linearis* spores and *A. bilimbi* branches. By employing a bio-guided isolation approach, we aim to identify bioactive compounds from these previously unexplored parts of the plants, thereby expanding the understanding of their potential therapeutic uses. This research will contribute to the growing body of knowledge on natural antioxidants and may offer valuable insights for the development of new pharmacological agents.

## 2. Materials and Methods

### 2.1. Chemicals

Thin-layer chromatography (TLC) was performed using precoated silica gel on aluminum plates [silica gel 60, F_254_ or RP–18 silica gel 60 F_254S_ (Merck, Darmstadt, Germany)]. The spots were visualized by heating after applying a 10% sulfuric acid solution. Silica gel column chromatography (CC) was conducted on a normal-phase silica gel 60 (40–63 µm, Merck). Reverse-phase silica gel CC was performed on a C18 silica gel 60 (23–40 µm, Merck). 1,1-Diphenyl-2-picrylhydrazyl (DPPH), gallic acid, and quercetin were obtained from Sigma-Aldrich Co. (St. Louis, MO, USA).

### 2.2. Plant Materials

Spores of *D. linearis* were collected in Binh Thuan province, Vietnam, in November 2022. The scientific name of the plant was authenticated by co-author Van-Son Dang. A voucher specimen (No. UE-P017A) was deposited in the VNM Herbarium, Institute of Tropical Biology, Vietnam Academy of Science and Technology.

The branches of *A. bilimbi* L. were collected in Uttaradit Province, Thailand, in October 2023. The specimens were identified by Asst. Prof. Dr. Kanit Wangwasit, Department of Biology, Faculty of Science, Mahasarakham University, Thailand, where a voucher specimen (K. Wangwasit 240807-1) was deposited.

### 2.3. Extraction and Isolation Procedure

The plant samples were dried in an oven at 55 °C for 48 h. Subsequently, the plant materials were ground, milled, and sieved using a 1 mm sieve. The obtained powder was weighed to determine the dry mass of each material before proceeding with the extraction process.

**Figure 1:**
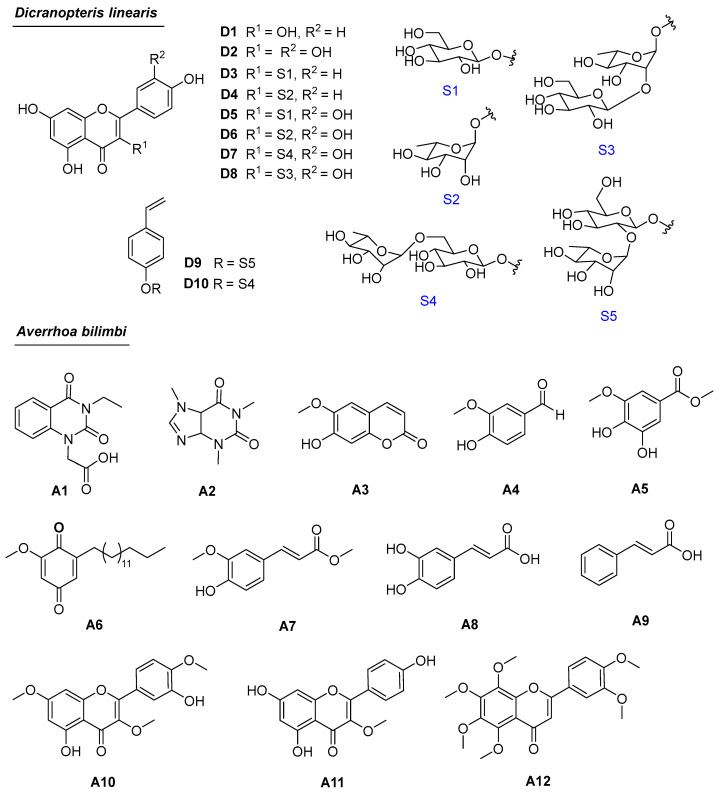
Chemical structures of compounds **D1**–**D10** and **A1**–**A12**.

The spores of *D. linearis* (300 g) were extracted with methanol (1 L × 10, each for 12 h) to yield a methanol extract (19 g). This extract underwent liquid–liquid extraction using *n*-hexane and ethyl acetate (1:1, *v*/*v*) to obtain the extracts of *n*-hexane (33 g, H), ethyl acetate-*n*-hexane (46 g, HEA), and ethyl acetate (82 g, EA), and a water-soluble extract (2.2 g). The EA extract was subsequently subjected to silica gel column chromatography (CC) employing a gradient of *n*-hexane–ethyl acetate (1:3–0:1, *v*/*v*), followed by methanol to yield four major fractions (EA1–EA4). Fractionation of EA3 (4.6 g) was performed using silica gel CC with a gradient of *n*-hexane–ethyl acetate (1:1–0.1, *v*/*v*) and subsequently methanol to afford five subfractions (EA3.1–EA3.5). Subfraction EA3.1 (1.2 g) was further purified by silica gel CC using a gradient of *n*-hexane–ethyl acetate (1.1:1–2:8, *v*/*v*) to yield subfractions X1–X3. Subfraction X2 (110 mg) was subjected to silica gel CC using *n*-hexane–ethyl acetate (1.1:1–2:8, *v*/*v*) to yield compounds **D1** (32.0 mg) and **D2** (15.0 mg). Subfraction EA3.4 (1.5 g) was loaded onto silica gel CC and eluted with ethyl acetate–methanol–water (gradient mode, 95:5:0.2–8:2:0.2, *v*/*v*/*v*) to obtain subfractions S1–S5. Subfraction S1 (210 mg) was purified by silica gel CC, eluting with ethyl acetate–methanol–water (95:5:0.2, *v*/*v*/*v*), to afford compounds **D3** (11.0 mg), **D4** (15.0 mg), and **D5** (15.0 mg). Compounds **D8** (3.0 mg) and **D9** (21.0 mg) were obtained after the separation of subfraction S2 (285 mg) by silica gel CC using ethyl acetate–methanol–water (9:1:0.2–8:2:0.2, *v*/*v*/*v*), while compounds **D6** (11.0 mg), **D7** (6.0 mg), and **D10** (2.0 mg) were isolated from subfraction S3 (180 mg) using the same solvent system. The detailed isolation procedure of compounds **D1**–**D10** (Figure 1) is provided in Scheme S1.

The branches of *A. bilimbi* (17 kg) were extracted with methanol (40 L × 3 each for 12 h) at room temperature, and the filtered solution was evaporated to afford a crude methanol extract (280 g). This extract underwent liquid–liquid partitioning using *n*-hexane and *n*-hexane–ethyl acetate (1:1, *v*/*v*), followed by ethyl acetate extraction to yield *n*-hexane (32.5 g, H), *n*-hexane–ethyl acetate (46.7 g, HEA), ethyl acetate (81.6 g, EA), and water-soluble (112 g) extracts, respectively. The HEA extract was subjected to silica gel CC using a gradient of *n*-hexane–ethyl acetate (2:1–0:1, *v*/*v*), followed by methanol, to afford nine major fractions (HEA1–HEA9). Fraction HEA3 (11.2 g) was further fractionated by Sephadex LH-20 CC using methanol as the mobile phase to yield three subfractions (HEA3.1–HEA3.3). Subfraction HEA3.2 (4.2 g) was isolated by silica gel CC using a gradient of *n*-hexane–ethyl acetate (4:1–2:8, *v*/*v*) to obtain three subfractions (R1–R3). Subfractions R2.1–R2.3 were obtained from subfraction R2 (510 mg) utilizing normal-phase silica gel CC as the stationary phase and *n*-hexane–ethyl acetate (4:1–2:8, *v*/*v*) as the eluent in a gradient mode. Subfraction R2.2 (45 mg) was further purified to afford compounds **A4** (2.8 mg), **A6** (3.0 mg), and **A7** (4.5 mg). Fraction HEA8 (7.6 g) was subjected to Sephadex LH-20 CC with methanol to yield four subfractions (HEA8.1–HEA8.4). Subfraction HEA8.2 (4.5 g) was purified by silica gel CC using a gradient of *n*-hexane–ethyl acetate (2:1–1:1, *v*/*v*) to afford subfractions S1–S4. Further purification of subfraction S1 (210 mg) by silica gel CC was conducted using chloroform–acetone–water (50:2.5:0.1, *v*/*v*/*v*) to afford compounds **A3** (8.0 mg) and **A5** (15 mg). Subfraction S2 (1.3 g) was subjected to RP-C18 CC using methanol–water (8:1, *v*/*v*) to obtain compounds **A1** (3.2 mg) and **A12** (4.5 mg). Purification of subfraction S3 (180 mg) by silica gel CC employing a gradient mode in a mixture of *n*-hexane–ethyl acetate (2:1–1:1, *v*/*v*) was carried out to obtain compounds **A10** (6.0 mg), **A11** (2.2 mg), and **A9** (11 mg). Subfraction S4 (210 mg) was loaded onto RP-C18 CC using methanol–water (3:1, *v*/*v*) to afford subfractions S4.1–S4.3. Compounds **A2** (5.0 mg) and **A8** (4.9 mg) were isolated from subfraction S4.1 (50 mg). An overview of the isolation procedure of compounds **A1**–**A12** (Figure 1) is provided in Scheme S2.

### 2.4. Structural Elucidation

Structural elucidation was conducted using NMR data and comparison with the literature. The NMR machine used was a Bruker Avance III (500 MHz) spectrometer with TMS as the internal standard. The Xevo G2 Quadrupole Time of Flight Mass Spectrometer was utilized to elucidate the molecular structures of a select number of compounds.

### 2.5. Total Phenolic Content (TPC)

The total phenolic content in the *A. bilimbi* and *D. linearis* extracts was quantified using the published method [26] with some modifications. Each extract was prepared at a concentration of 10 g/L. A volume of 0.1 mL of each extract was mixed with 0.5 mL of Folin–Ciocalteu reagent (diluted with water with the ratio of 1:10, *v*/*v*) for 5 min. Next, 1.5 mL of sodium carbonate (7.5%, *w*/*v*) were added. The tube content was incubated for 40 min in the dark, and the absorbance was read at 760 nm. Different concentrations of gallic acid ranging from 0.45 to 28.8 µg/mL were prepared. The TPC of samples was estimated from the standard curve of gallic acid (20–200 µg/mL).

### 2.6. Total Flavonoid Content (TFC)

The total flavonoid content in the *A. bilimbi* and *D. linearis* extracts was evaluated following the method described previously [26] with some modifications. Each extract was prepared at a concentration of 10 g/L. Concentrations of 1 g/L, 1 M, and 1 M of agents AlCl_3_, NaNO_2_, and NaOH were prepared, respectively. A volume of 1 mL of each extract was mixed with 4 mL H_2_O and then 0.3 mL NaNO_2_ in the dark for 5 min. Next, 0.3 mL AlCl_3_ and 2 mL NaOH and then 2.4 mL were added to the mixture. The mixture was allowed to stay in the dark for 30 min. Finally, the absorbance was measured at 510 nm. The TFC was evaluated based on the calibration curve of a standard compound, quercetin (20–200 µg/mL).

### 2.7. DPPH Free Radical Scavenging Activity

The scavenging activity of extracts and compounds towards free radicals 1,1-diphenyl-2-picrylhydrazyl (DPPH) was assessed using the methodology outlined by Ponnusamy et al. [4]. The DPPH 0.2 mM solution in methanol was freshly prepared before the test. For the test samples, 100 µL DPPH 0.2 mM were mixed with *A. bilimbi* and *D. linearis* extracts and selected compounds in a 96-well plate and incubated at room temperature in the dark for 30 min. The reference samples contained only DPPH solution, and the prepared blank samples did not contain extracts or compounds. The absorbance was measured at 517 nm, and the radical scavenging activity was calculated using the following equation:

$$\%DPPH\;Radical\;scavenging\;activity=\left(1-\frac{A_{Sample}-A_{Sample\;(Blank)}}{A_{Reference}-A_{Reference\;(Blank)}}\right)*100$$

### 2.8. ABTS Free Radical Scavenging Ability

The 2,2-azino-di-[3-ethylbenzthiazoline sulfonate (ABTS) free radical scavenging ability of extracts and compounds was determined according to Jiang et al. [27]. The solution containing 4.5 mL of ABTS (7 mM) and 4.5 mL of K_2_S_2_O_8_ (2.45 mM) in ammonium acetate buffer (pH 4.5) was kept away from light for 16 h to prepare the ABTS solution. Then, 3.0 mL of the ABTS solution were added to 1.5 mL of compounds and extracts (at a starting concentration of 200 µg/mL) and allowed to react for 6 min in brown tubes. The absorbance was measured at 734 nm, and the radical scavenging activity was calculated using the following equation:

$$\%ABTS\;Radical\;scavenging\;activity=\left(1-\frac{A_{Sample\;}-A_{Sample\;(Blank)\;}}{A_{Reference}-A_{Reference\;(Blank)\;}}\right)*100$$

### 2.9. Statistical Analysis

All samples were analyzed in triplicate. Statistical comparisons were performed using the one-way analysis of variance (ANOVA) test. All the data were expressed as mean values with the standard deviations (mean ± S.D.).

### 2.10. HPLC Analysis for Extracts and Selected Compounds

**Figure 2:**
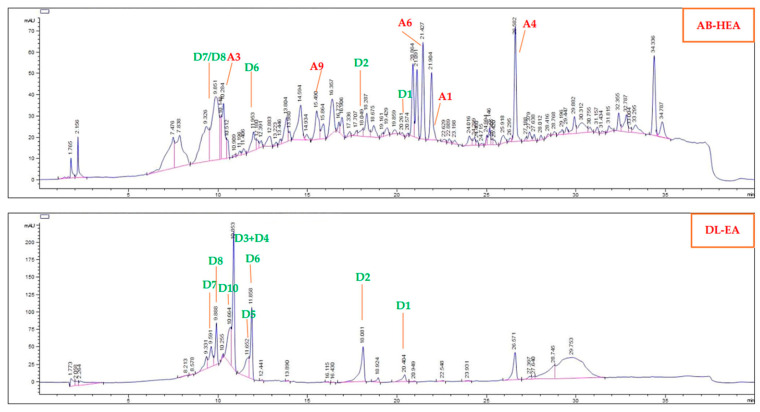
HPLC chromatograms of the *A. bilimbi* HEA extract and the *D. linearis* EA extract, showing the presence of compounds **D1**–**D10**, **A3**, **A4**, **A6**, **A9**, and the new compound **A1**.

The extracts and selected compounds were applied to HPLC-DAD analysis. Compounds and extracts were injected separately through a Luna Phenomenex C18 column (150 mm × 4.6 mm, 5 µm). The mobile phase consisted of (H_2_O/0.1% HCOOH) as solvent A and (CH_3_CN/0.1% HCOOH) as solvent B with a gradient of 40–95% B for 45 min and 95–100% B for 5 min. The flow rate was 1 mL/min, and 10 µL of each sample were injected. The HPLC chromatogram showed the presence of compounds in the corresponding extracts (Figure 2). The purities of bioactive compounds **D3**, **D8**, **D10**, **A3**, and **A6** were determined by the same HPLC method. The chromatograms (Figure S6A–E) are presented in the Supplementary Materials.

## 3. Results

### 3.1. Analysis of Total Phenolic and Flavonoid Contents, and Antioxidant Activity in A. bilimbi and D. linearis Extracts

The total phenolic content (TPC), total flavonoid content (TFC), and antioxidant activities of the extracts from *D. linearis* spores and *A. bilimbi* branches were determined, as summarized in Table 1.

For *D. linearis* spores, the EA extract showed the highest total phenolic content (TPC) of 120.13 ± 0.04 mg GAE/g and total flavonoid content (TFC) of 21.94 ± 0.30 mg QE/g, along with strong antioxidant activity. The extract exhibited 96.3 ± 0.3% inhibition (IC_50_ of 39.4 ± 0.3 µg/mL) for DPPH and 86.0 ± 0.2% inhibition (IC_50_ of 88.9 ± 0.7 µg/mL) for ABTS.

For *A. bilimbi* branches, the HEA extract exhibited the highest TPC of 165.21 ± 0.24 mg GAE/g and TFC of 26.20 ± 0.01 mg QE/g. The extract showed 89.6 ± 0.7% inhibition for DPPH, with an IC_50_ of 39.7 ± 1.9 µg/mL, and 44.4 ± 0.2% inhibition for another assay (IC_50_ of 130.8 ± 1.4 µg/mL).

### 3.2. Phytochemical Investigations of A. bilimbi and D. linearis

From the EA extract of *D. linearis* spores, eight flavonoids (**D1**–**D8**) and two phenolic glycosides (**D9** and **D10**) were isolated, including kaempferol (**D1**) [10], quercetin (**D2**) [10], astragalin (**D3**) [28], afzelin (**D4**) [29], isoquercetin (**D5**) [30], quercitrin (**D6**) [31], rutin (**D7**) [32], kaempferol 3-*O*-[3-D-glucopyranosyl)-*α*-L-rhamnopyranoside (**D8**) [33], 4-vinyl-phenol-1-*O*-[*α*-L-rhamno(1→2)-*β*-D-glucopyranoside (**D9**) [4], and 4-vinyl-phenol-1-*O*-[*α*-L-rhamno(1→6)-*β*-D-glucopyranoside (**D10**) [34]. The chemical structures of all isolated compounds were elucidated through nuclear magnetic resonance (NMR) spectroscopy, high-resolution mass data in both negative and positive modes, and comparison with the corresponding literature data. The NMR data of compounds **D3**–**D6** and **D8**–**D10** are presented below.

Astragalin (**D3**): ^1^H-NMR (500 MHz, DMSO-*d_6_*, *δ* ppm, *J* in Hertz): 12.60 (1H, *brs*, 5-OH), 8.04 (2H, *d*, *J* = 9.0 Hz, H-2′, H-6′), 6.88 (2H, *d*, *J* = 9.0 Hz, H-3′, H-5′), 6.42 (1H, *d*, *J* = 2.0 Hz, H-8), 6.20 (1H, *d*, *J* = 2.0 Hz, H-6), 5.45 (1H, *d*, *J* = 7.5 Hz, H-1″), 3.57 (1H, *m*, H-6″a), 3.20–3.40 (4H, *overlap*, H-2″, H-3″, H-4″, H-5″), 3.19 (1H, *m*, H-6″b) [28].

Afzelin (**D4**): ^1^H-NMR (500 MHz, DMSO-*d_6_*, *δ* ppm, *J* in Hertz): 12.64 (1H, *s*, 5-OH), 10.87 (1H, *s*, 7-OH), 9.74 (1H, *s*, 3′-OH), 9.19 (1H, *s*, 4′-OH), 7.76 (2H, *d*, *J* = 8.5 Hz, H-2′, H-6′), 6.92 (2H, *d*, *J* = 8.5 Hz, H-3′, H-5′), 6.41 (1H, *s*, H-8), 6.21 (1H, *brs*, H-6), 5.30 (1H, *s*, H-1″), 3.09–3.15 (4H, *m*, H-2″, H-3″, H-4″, H-5″), 0.80 (1H, *d*, *J* = 6.0 Hz, H-6″). ^13^C-NMR (125 MHz, DMSO-*d*_6_, *δ* ppm): 177.7 (C-4), 164.2 (C-5), 161.3 (C-7), 160.0 (C-2), 157.2 (C-9), 148.3 (C-3′), 144.7 (C-4′), 134.2 (C-3), 121.9 (C-1′), 120.9 (C-6′), 120.0 (C-2′), 116.0 (C-5′), 104.1 (C-10, C-1″), 98.7 (C-6), 93.7 (C-8), 71.1 (C-4″), 70.6 (C-2″), 70.3 (C-3″), 70.0 (C-5″), 17.4 (C-6″) [29]. HRESIMS (positive mode) *m/z* 449.1091 [M+H]^+^ (cald. for C_21_H_21_O_11_ 449.1081).

Isoquercetin (**D5**): ^1^H-NMR (500 MHz, DMSO-*d_6_*, *δ* ppm, *J* in Hertz): 12.64 (1H, *s*, 5-OH), 10.87 (1H, *s*, 7-OH), 9.74 (1H, *s*, 3′-OH), 9.19 (1H, *s*, 4′-OH), 7.66 (1H, *d*, *J* = 8.5 Hz, H-6′), 7.52 (1H, *s*, H-2′), 6.81 (1H, *d*, *J* = 8.5 Hz, H-5′), 6.40 (1H, *s*, H-8), 6.20 (1H, *s*, H-6), 5.37 (1H, *d*, *J* = 8.0 Hz, H-1″), 5.29 (1H, *brs*, 2″-OH), 5.14 (1H, *brs*, 3″-OH), 4.87 (1H, *brs*, 4″-OH), 4.44 (1H, *brs*, 6″-OH), 3.56 (2H, *m*, H-6″), 3.24 (1H, *m*, H-2″), 3.23 (1H, *m*, H-3″), 3.08 (1H, *m*, H-4″), 3.07 (1H, *m*, H-5″). ^13^C-NMR (125 MHz, DMSO-*d*_6_, *δ* ppm): 177.3 (C-4), 164.0 (C-5), 161.1 (C-7), 156.1 (C-2, C-9), 148.3 (C-3′), 144.7 (C-4′), 133.3 (C-3), 121.9 (C-1′), 120.9 (C-6′), 116.0 (C-5′), 115.0 (C-2′), 103.8 (C-10), 101.6 (C-1″), 98.5 (C-6), 75.7 (C-5″), 73.0 (C-3″), 71.0 (C-2″), 67.8 (C-4″), 60.0 (C-6″) [30].

Quercitrin (**D6**): ^1^H-NMR (500 MHz, DMSO-*d_6_*, *δ* ppm, *J* in Hertz): 12.64 (1H, *s*, 5-OH), 10.87 (1H, *s*, 7-OH), 9.74 (1H, *s*, 3′-OH), 9.19 (1H, *s*, 4′-OH), 7.66 (1H, *d*, *J* = 8.5 Hz, H-6′), 7.52 (1H, *s*, H-2′), 6.92 (1H, *d*, *J* = 8.5 Hz, H-5′), 6.38 (1H, *d*, *J* = 2.0 Hz, H-6), 6.21 (1H, *d*, *J* = 1.5 Hz, H-8), 5.35 (1H, *s*, H-1″), 4.21 (1H, *m*, H-2″), 3.23 (1H, *m*, H-3″), 3.08 (1H, *m*, H-4″), 3.07 (1H, *m*, H-5″), 0.95 (1H, *d*, *J* = 6.0 Hz, H-6″). ^13^C-NMR (125 MHz, DMSO-*d*_6_, *δ* ppm): 179.6 (C-4), 165.8 (C-7), 163.1 (C-5), 159.4 (C-2), 158.5 (C-9), 149.7 (C-4′), 146.3 (C-3′), 136.0 (C-3), 122.9 (C-6′), 122.8 (C-1′), 116.9 (C-5′), 116.4 (C-2′), 105.9 (C-10), 103.5 (C-1″), 99.9 (C-6), 94.8 (C-8), 73.2 (C-4″), 73.0 (C-5″), 72.0 (C-3″), 71.9 (C-2″), 17.6 (C-6″) [31].

Kaempferol 3-*O*-*β*-D-glucopyranoside-7-*O*-*α*-L-rhamnopyranoside (**D8**): ^1^H-NMR (500 MHz, methanol-*d*_4_, *δ* ppm, *J* in Hertz): 7.78 (2H, *d*, *J* = 8.5 Hz, H-2′, H-6′), 6.96 (2H, *d*, *J* = 9.0 Hz, H-3′, H-5′), 6.41 (1H, *d*, *J* = 2.0 Hz, H-8), 6.23 (1H, *d*, *J* = 2.0 Hz, H-6), 5.72 (1H, *d*, *J* = 1.0 Hz, H-1″), 5.14 (1H, *t*, *J* = 9.0 Hz, H-4″), 4.42 (1H, *d*, *J* = 8.0 Hz, H-1‴), 4.29 (1H, *dd*, *J* = 3.5, 1.5 Hz, H-2″), 3.83 (1H, *dd*, *J* = 10.5, 3.5 Hz, H-3″), 3.70 (1H, *m*, H-6″). ^13^C-NMR (125 MHz, methanol-*d*_4_, *δ* ppm): 179.6 (C-4), 166.0 (C-7), 163.2 (C-5), 161.7 (C-4′), 159.5 (C-2), 158.6 (C-9), 136.5 (C-3), 132.0 (C-2′, C-6′), 122.6 (C-1′), 116.6 (C-3′, C-5′), 107.1 (C-1‴), 106.0 (C-10), 102.6 (C-1″), 100.0 (C-6), 94.9 (C-8), 82.6 (C-2″), 77.9 (C-3‴, C-5‴), 75.3 (C-2‴), 73.4 (C-4″), 72.0 (C-5″), 71.8 (C-3″), 70.9 (C-4‴), 62.4 (C-6‴), 17.6 (C-6″) [33]. HRESIMS (positive mode) *m/z* 593.1506 [M-H_2_O+H]^+^ (cald. for C_27_H_29_O_15_ 593.1533).

4-Vinyl-phenol-1-*O*-[*α*-L-rhamno(1→2)-*β*-D-glucopyranoside (**D9**): ^1^H-NMR (DMSO-*d*_6_, 500 MHz) 7.40 (2H, *d*, *J* = 8.5 Hz, H-3, H-5), 6.99 (2H, *d*, *J* = 8.5 Hz, H-2, H-6), 6.67 (1H, *dd*, *J* = 18.0, 11.0 Hz, H-7), 5.70 (1H, *d*, *J* = 18.0 Hz, H-8a), 5.16 (1H, *d*, *J* = 7.0 Hz, H-1′), 5.14 (1H, *d*, *J* = 11.5 Hz, H-8b), 4.75 (1H, *d*, *J* = 5.5 Hz, 4″-OH), 4.55 (1H, *brs,* H-1″), 3.85 (1H, *d*, *J* = 10.5 Hz, H-6′a), 3.77 (1H, *m*, H-4′), 3.71 (1H, *m*, H-2′), 3.67 (1H, *m*, H-3′), 3.61 (3H, *m,* H-6′b, H-2″, H-3″), 3.60 (2H, *m*, H-5′, H-5″), 3.30 (1H, *m*, H-4″), 1.11 (1H, *d*, *J* = 6.0 Hz, H-6″). ^13^C-NMR (DMSO-*d*_6_, 125 MHz) 157.2 (C-1), 136.1 (C-7), 131.2 (C-4), 127.2 (C-3, C-5), 116.4 (C-2, C-6), 112.4 (C-8), 100.7 (C-1″), 100.5 (C-1′), 77.8 (C-3″), 76.6 (C-2′), 72.0 (C-4″), 70.7 (C-3′), 70.4 (C-2″), 70.0 (C-5′), 68.4 (C-5″), 66.7 (C-4′), 61.0 (C-6′), 17.9 (C-6″) [4]. HRESIMS (negative mode) *m/z* 473.1648 [M+HCO_2_H-H]^−^ (cald. for C_20_H_29_O_10_ 473.1659).

4-Vinyl-phenol-1-*O*-[*α*-L-rhamno(1→6)-*β*-D-glucopyranoside (**D10**): ^1^H-NMR (Methanol-*d*_4_, 500 MHz) 7.37 (2H, *d*, *J* = 9.0 Hz, H-3, H-5), 7.03 (2H, *d*, *J* = 8.5 Hz, H-2, H-6), 6.64 (1H, *overlap*, H-7), 5.66 (1H, *dd*, *J* = 17.5, 0.5 Hz, H-8a), 5.11 (1H, *dd*, *J* = 11.0, 1.0 Hz, H-8b), 4.85 (1H, *overlap*, H-1′), 4.70 (1H, *d*, *J* = 2.0, H-1″), 4.03 (1H, *m*, H-6′a), 3.85 (1H, *m*, H-2″), 3.71 (1H, *m*, H-3″), 3.69 (1H, *m*, H-4′), 3.66 (1H, *m*, H-5″), 3.60 (1H, *m*, H-6′b), 3.45 (1H, *m*, H-2′), 3.44 (1H, *m*, H-5′), 3.36 (1H, *m*, H-4″), 3.33 (1H, *m*, H-3′), 1.20 (1H, *d*, *J* = 6.0 Hz, H-6″). ^13^C-NMR (Methanol *d*_4_, 125 MHz) 158.8 (C-1), 137.5 (C-7), 133.6 (C-4), 128.3 (C-3, C-5), 117.8 (C-2, C-6), 112.5 (C-8), 102.3 (C-1′), 102.2 (C-1″), 75.1 (C-2′), 74.9 (C-3′), 74.0 (C-4″), 72.4 (C-4′, C-3″), 72.2 (C-2″), 71.6 (C-5′), 69.9 (C-5″), 67.9 (C-6′), 17.9 (C-6″) [34]. HRESIMS (negative mode) *m/z* 473.1699 [M+HCO_2_H-H]^−^ (cald. for C_20_H_29_O_10_ 473.1659).

From the HEA extract of *A. bilimbi*, twelve compounds were isolated, including 2-(3-ethyl-2,4-dioxo-3,4-dihydroquinazolin-1(2*H*)-yl)acetic acid (**A1**) [35,36], caffeine (**A2**) [37], scopoletin (**A3**) [38], vanilin (**A4**), 3-methoxygallate methyl (**A5**), 2-dehydroxy-5-*O*-methylembelin (**A6**) [39], methyl ferulate (**A7**) [40], 3,4-dihydroxycinnamic acid (**A8**), cinnamic acid (**A9**), ayanin (**A10**) [41], 3-*O*-methoxykaempferol (**A11**) [42], and nobiletin (**A12**) [43]. The chemical structures of all isolated compounds were elucidated through NMR spectroscopy and comparison with the corresponding literature data. The NMR data of compounds **A1**-**A3**, **A7**, **A9, A10**, and **A12** are presented in the Supplementary Materials.

2-(3-Ethyl-2,4-dioxo-3,4-dihydroquinazolin-1(2*H*)-yl)acetic acid (**A1**): ^1^H-NMR (acetone-*d*_6_, 500 MHz) 8.13 (1H, *dd*, *J* = 8.0, 1.5 Hz, H-8), 7.77 (1H, *ddd*, *J* = 8.5, 7.0, 1.5 Hz, H-6), 7.49 (1H, *d*, *J* = 8.5 Hz, H-5), 7.45 (1H, *ddd*, *J* = 8.0, 7.0, 1.0 Hz, H-7), 4.21 (2H, *q*, *J* = 7.0 Hz, H-1′), 4.12 (2H, *s*, H-2′), 1.38 (3H, *t*, *J* = 7.0 Hz, H-1″), 1.27 (3H, *t*, *J* = 7.0 Hz, H-2″″)**.** ^13^C-NMR (acetone-*d*_6_, 125 MHz) 169.0 (C-2″), 161.3 (C-1), 156.7 (C-3), 147.0 (C-10), 135.2 (C-6), 127.5 (C-8), 126.9 (C-7), 126.7 (C-5), 119.7 (C-9), 40.5 (C-1′), 34.9 (C-2′), 14.5 (C-1″).

Caffeine (**A2**): ^1^H-NMR (CDCl_3_, 500 MHz) 7.71 (1H, *s*, H-6), 3.99 (3H, *s*, H-3′), 3.59 (3H, *s*, H-2′), 3.41 (3H, *s*, H-1′). ^13^C-NMR (CDCl_3_, 125 MHz) 155.3 (C-8), 151.4 (C-1), 148.7 (C-3), 141.1 (C-9), 107.4 (C-6), 33.6 (C-3′), 29.7 (C-2′), 27.9 (C-1′) [37].

Scopoletin (**A3**): ^1^H-NMR (acetone-*d*_6_, 500 MHz) 7.85 (1H, *d*, *J* = 9.5 Hz, H-3), 7.20 (1H, *s*, H-5), 6.80 (1H, *s*, H-8), 6.18 (1H, *d*, *J* = 9.5 Hz, H-2), 3.90 (3H, *s*, H-10). ^13^C-NMR (acetone-*d*_6_, 125 MHz) 160.8 (C-1), 151.9 (C-7), 146.0 (C-6), 144.7 (C-3), 115.8 (C-2), 113.4 (C-4), 110.1 (C-5), 103.8 (C-8), 56.8 (C-10) [38].

2-Dehydroxy-5-*O*-methylembelin (**A6**): ^1^H-NMR (CDCl_3_, 500 MHz) 6.48 (1H, *s*, H-5), 5.87 (1H, *s*, H-3), 3.82 (3H, *s*, 2-OMe), 2.42 (2H, *m*, H-1′), 1.50 (2H, *m*, H-2′), 1.25 (24H, *m*, H-3′–H-14′), 0.88 (3H, *s*, H′-15) [39].

Methyl ferulate (**A7**): ^1^H-NMR (CDCl_3_, 500 MHz) 7.63 (1H, *d*, *J* = 16.0 Hz, H-7), 7.08 (1H, *dd*, *J* = 8.0, 1.5 Hz, H-6), 7.03 (1H, *d*, *J* = 1.5 Hz, H-2), 6.93 (1H, *d*, *J* = 8.0 Hz, H-5), 6.30 (1H, *d*, *J* = 16.0 Hz, H-8), 3.93 (3H, *s*, H-11), 3.80 (3H, *s*, H-10).

Cinnamic acid (**A8**): ^1^H-NMR (CDCl_3_, 500 MHz) 7.79 (1H, *d*, *J* = 16.0 Hz, H-3), 7.54–7.56 (4H, *m*, H-2′, H-6′), 7.39–7.42 (9H, *m*, H-3′, H-4′, H-5′), 6.46 (1H, *d*, *J* = 16.0 Hz, H-2).

Ayanin (**A10**): ^1^H-NMR (acetone-*d*_6_, 500 MHz) 12.75 (1H, *s*, 5-OH), 8.49 (1H, *s*, 3′-OH), 7.72–7.69 (1H, *m*, H-2′), 7.71 (1H, *dd*, *J* = 8.5, 2.0 Hz, H-6′), 7.01 (1H, *d*, *J* = 8.5 Hz, H-5′), 6.67 (1H, *d*, *J* = 2.0 Hz, H-8), 6.32 (1H, *d*, *J* = 2.0 Hz, H-6), 3.94 (3H, *s*, 4′-OMe), 3.91 (3H, *s*, 7-OMe), 3.89 (3H, *s*, 3-OMe).

3-*O*-Methylkaemperol (**A11**): ^1^H-NMR (DMSO-*d*_6_, 500 MHz) 11.72 (1H, *s*, 5-OH), 7.96 (2H, *d*, *J* = 9.0 Hz, H-2′, H-6′), 6.86 (2H, *d*, *J* = 8.5 Hz, H-3′, H-5′), 6.27 (H, *d*, *J* = 2.0 Hz, H-8), 6.22 (H, *d*, *J* = 2.0 Hz, H-6), 3.77 (3H, s, 3-OMe).

Nobiletin (**A12**): ^1^H-NMR (CDCl_3_, 500 MHz) 7.58 (1H, *dd*, *J* = 8.5, 2.5 Hz, H-6′), 7.42 (1H, *d*, *J* = 2.5 Hz, H-2′), 7.0 (1H, *d*, *J* = 8.5 Hz, H-5′), 6.62 (1H, *s*, H-3), 4.10 (3H, *s*, 8-OMe), 4.02 (3H, *s*, 7-OMe), 3.96 (6H, *s*, 3′-OMe, 4′-OMe), 3.95 (6H, *s*, 5-OMe, 6-OMe).

### 3.3. Antioxidant Activities of Compounds **D3**–**D5**, **D8**–**D10**, and **A1**–**A11**

Compounds **D3**–**D5**, **D8**–**D10**, and **A1**–**A11** were evaluated for their DPPH and ABTS free radical scavenging activities. The results are shown in Table 2. The free radical DPPH scavenging activity of the selected compounds, as indicated by their IC_50_ values, varied significantly. Among the tested compounds, compound **A3** demonstrated the strongest DPPH antioxidant activity, with an IC_50_ of 7.1 ± 0.1 µg/mL, followed by **D8** (7.3 ± 0.8 µg/mL) and **D3** (14.1 ± 1.4 µg/mL). Compounds **D10** and **A6** also exhibited notable activity, with IC_50_ values of 12.3 ± 0.5 and 39.7 ± 1.9 µg/mL, respectively. Other compounds (**A1**, **A2**, **A4**, **A5**, and **A7–A11**) had IC_50_ values greater than 100 µg/mL, indicating no antioxidant activity. For the free radical ABTS scavenging activity, compounds **D3**, **D4**, **D8**, **A3**, **A6**, and **A9** showed moderate activity, with IC_50_ values ranging from 14.8 to 133.5 µg/mL (Table 2). Among them, compound **A3** exhibited the highest ABTS antioxidant activity, with an IC_50_; of 14.8 ± 0.1 µg/mL. Other compounds were weak or inactive.

### 3.4. HPLC-DAD Analysis of A. bilimbi and D. linearis Extracts

HPLC-DAD analysis was applied to *A. bilimbi* and *D. linearis* extracts, and the results are shown in Figure 2, Figure S4, and Figure S5. Figure 2 illustrates the presence of compounds **D1–D10**, **A3**, **A4**, **A6**, **A9**, and the new compound **A1** in the *A. bilimbi* HEA extract and the *D. linearis* EA extract. Figures S4 and S5 display the HPLC chromatograms of different extracts derived from *A. bilimbi* and *D. linearis*.

## 4. Discussion

As shown in Table 1, extracts prepared from the spores of *D. linearis* exhibited high free radical scavenging activity. Previous studies have indicated that *D. linearis* leaves possess potent antioxidant activity, which is attributed to high TPC and DPPH scavenging activity [6,11,12] (Table 3). A literature review suggests that polar extracts demonstrate stronger activity than the less polar CHCl_3_ extract, a result that is consistent with our findings. Ponnusamy and co-workers indicated that highly polar fractions exhibit significant antioxidant activity in DPPH and FRAP assays [4]. However, little is known about the TFC and antioxidant ABTS activity in *D. linearis*. Chemical analysis suggests that flavonoids, flavonoid glycosides, and phenylethanoid glycosides contribute to the strong antioxidant activity of *D. linearis*.

Compounds such as kaempferol (**D1**), quercetin (**D2**), afzelin (**D4**), isoquercetin (**D5**), and 4-vinyl-phenol-1-*O*-[*α*-L-rhamno(1→2)-*β*-D-glucopyranoside] (**D9**) have been previously reported in the leaves of *D. linearis* from Malaysia [4]. This study reports compounds **D3** and **D6**–**D10** in *D. linearis* for the first time. Flavonoids are generally considered to be key antioxidant components [44], with quercetin, kaempferol, and rutin being well-known examples. Compound **D8** exhibited stronger activity than analogs **D3–D5**, highlighting the importance of the second sugar unit (Table 2). This observation is consistent with findings that rutin has stronger activity compared to quercetin and kaempferol. Compound **D3** is stronger than **D4** and **D5**, indicating that either the 3′-OH group (in **D5**) or the L-rhamnose unit decreases the activity. Even though compounds **D9** and **D10** have two sugar units, the activity of **D10** is stronger than **D9**, suggesting that the linkage between the two sugars plays an important role in activity.

Although compound **D9** has been previously identified in *D. linearis* leaves [4], its biological activity has not been reported. 4-Vinyl-phenol-1-*O*-[*α*-L-rhamno(1→6)-*β*-D-glucopyranoside] (**D10**) was isolated from *Asplenium trichomanes* and has shown moderate estrogenic activity [39].

Several recent studies have reported on TPC, TFC, and DPPH scavenging activity in the fruits and leaves of *A. bilimbi* [14,18,21,23]. However, little is known about the DPPH and ABTS scavenging activities of *A. bilimbi* branches. As shown in Table 1, all extracts prepared from *A. bilimbi* exhibited higher TPC and TFC than those from *D. linearis*.

**Figure 3:**
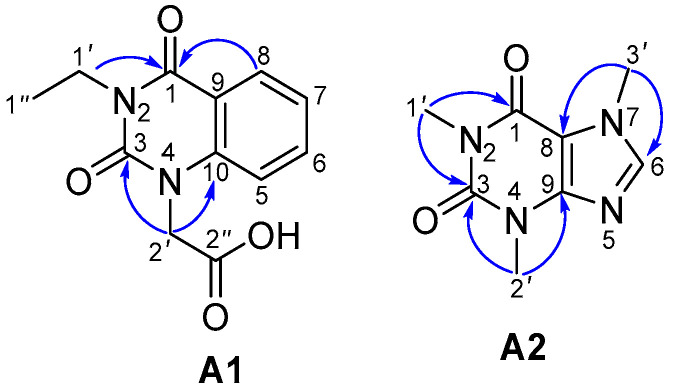
Selected heteronuclear multiple bond correlations of compounds **A1** and **A2**.

Compounds **A1**–**A7** and **A9**–**A11** were reported in *A. bilimbi* for the first time. Although compound **A1** was synthetically prepared [35,36], it is a new natural compound. Its NMR spectra are provided in the Supplementary Materials (Figure S2A–G). Selected HMBC correlations of **A1** and **A2** are shown in Figure 3. To date, 17 compounds have been isolated from *A. bilimbi* sources (Figure S1) [16,18]. Among them, aurantiamide benzoate is an amide-type alkaloid (Figure S1). Compounds **A1** and **A2** represent a new alkaloid type found in the *Averrhoa* genus. A comparison of NMR data for compounds **A1**, **A2**, and other analogs is provided in Tables S14 and S15.

Although the HEA extract was the most active in terms of DPPH/ABTS scavenging activities, most isolated compounds showed weak activities, except for **A3** and **A6**. Of these, scopoletin (**A3**) was the most active compound, with an IC_50_ value of 7.1 ± 0.1 µg/mL. This finding aligns with those reported in the literature [45,46]. 2-Dehydroxy-5-*O*-methylembelin (**A6**), previously found in *A. carambola* [47], has limited biological data available. The antioxidant potential of this compound might be attributed to its quinone skeleton [48]. Scopoletin and 2-dehydroxy-5-*O*-methylembelin likely contribute to the activity of the HEA extract.

Although compounds **D8**–**D10**, **A1**, and **A6** are known, little is known about their biological activity. Flavonoid analogs **D1**–**D7**, which share a similar structure to **D8**, have been reported as promising candidates for anti-diabetic and antimicrobial drugs [28,29,49]. Compound **A5** exhibited moderate alpha-glucosidase inhibition and antimicrobial activity against various bacterial strains [50]. Further anti-diabetic and antimicrobial assays should be conducted on compounds **D8**–**D10**, **A1**, and **A6**.

HPLC-DAD analysis indicated that the EA extract of *D. linearis* contains compounds **D1**–**D10** in high concentrations (Figure 2). The presence of antioxidant compounds **D3**–**D10** in all crude, HEA, EA, and water-soluble extracts of *D. linearis* suggests that these are major compounds and could serve as biomarkers for *D. linearis* spores. Similarly, HPLC-DAD analysis revealed the presence of bioactive compounds **A3**, **A6**, and **A9** in the HEA extract of *A. bilimbi* branches. The most active compound (**A3**) was not detected in the H and EA extracts of *A. bilimbi*, which might explain the weak activity of these extracts.

## 5. Conclusions

This study successfully applied bio-guided isolation based on antioxidant activities to *D. linearis* spores and *A. bilimbi* branches, leading to the isolation of 22 compounds (**D1**–**D10** and **A1**–**A12**). To the best of our knowledge, compound **A1** was identified as a new natural compound. Compound **A3** demonstrated the most potent antioxidant activities (IC_50_ values of 7.1 ± 0.1 µg/mL for DPPH, and 14.8 ± 0.1 µg/mL for ABTS, respectively). These findings are significant, as they expand the understanding of the bioactive potential of these previously unexplored plant parts. The results suggest that *D. linearis* spores and *A. bilimbi* branches could serve as valuable sources of natural antioxidants, offering promising prospects for their application in developing therapeutic agents aimed at combating oxidative stress-related diseases such as cancer, diabetes, and cardiovascular conditions. This research emphasizes the importance of continuing to explore underutilized plant resources, as they may contribute to the discovery of novel compounds with potent therapeutic properties. However, this study did not investigate the in vivo efficacy of the compounds or their potential side effects. Further research is needed to evaluate the full therapeutic potential of these compounds.

## Figures and Tables

**Table 1:** Results of total phenolic content (TPC), total flavonoid content (TFC), and antioxidant activities of *A. bilimbi* and *D. linearis* extracts.

Biosource	Extract	TPC	TFC	DPPH		ABTS	
		(mg GAE/g)	(mg QE/g)	%I	IC_50_ (µg/mL)	%I	IC_50_ (µg/mL)
*D. linearis* spores	Crude	62.35 ^f^ ± 0.04	8.09 ^g^ ± 0.10	98.8 ^a^ ± 0.2	35.5 ^b^ ± 0.2	69.8 ± 0.4 ^d^	132.2 ± 0.7 ^a^
	HEA	51.09 ^g^ ± 0.03	15.53 ^f^ ± 0.08	41.0 ^e^ ± 0.3		12.0 ± 0.3 ^c^	
	EA	120.13 ^d^ ± 0.04	21.94 ^c^ ± 0.30	96.3 ^b^ ± 0.3	39.4 ^a^ ± 0.3	86.0 ± 0.2 ^b^	88.9 ± 0.7 ^a^
*A. bilimbi* branches	Crude	213.34 ^a^ ± 0.06	31.56 ^a^ ± 0.03	38.9 ^f^ ± 1.2		37.2 ± 0.1 ^a^	539.5 ± 15.2 ^c^
	*n*-Hexane	125.30 ^c^ ± 0.01	19.12 ^d^ ± 0.03	18.2 ^g^ ± 1.4		-	-
	HEA	165.21 ^b^ ± 0.24	26.20 ^b^ ± 0.01	89.6 ^c^ ± 0.7	39.7 ^a^ ± 1.9	44.5 ± 0.2 ^a^	130.8 ± 1.4 ^b^
	EA	112.72 ^e^ ± 0.21	18.38 ^e^ ± 0.19	48.9 ^d^ ± 0.7		12.8 ± 0.4 ^b^	-
Ascorbic acid (positive control)					2.1 ^c^ ± 0.2		4.0 ± 0.1 ^b^

% Inhibition at 200 µg/mL. a, b, c, d, e, f, and g show statistically significant differences at the 95.0% confidence level between data in the same column (*p* < 0.05), with a > b > c > d > e > f > g.

**Table 2:** Free radical scavenging activities of selected compounds **D3**–**D5**, **D8**–**D10**, and **A1**–**A11**.

Compound	DPPH	ABTS
	IC_50_ (µg/mL)	IC_50_ (µg/mL)
**D3**	14.1 ^d^ ± 1.4	30.4 ± 0.1 ^e^
**D4**	48.0 ^a^ ± 1.9	133.5 ± 1.2 ^a^
**D5**	27.4 ^c^ ± 2.1	>300
**D8**	7.3 ± 0.8	26.9 ± 0.1 ^f^
**D9**	39.4 ^b^ ± 0.3	-
**D10**	12.3 ^d^ ± 0.5	-
**A1**	>100	-
**A2**	>100	-
**A3**	7.1 ^e^ ± 0.1	14.8 ± 0.1 ^g^
**A4**	>100	>300
**A5**	>100	>300
**A6**	39.7 ^a^ ± 1.9	37.3 ± 0.1 ^d^
**A7**	>100	>300
**A8**	>100	>300
**A9**	>100	131.4 ± 0.7 ^b^
**A10**	>100	>300
**A11**	>100	>300
Ascorbic acid (positive control)	2.1 ^b^ ± 0.2	4.0 ± 0.1 ^h^

a, b, c, d, e, f, g, and h show statistically significant differences at the 95.0% confidence level between data in the same column (*p* < 0.05), with a > b > c > d > e > f > g > h.

**Table 3:** Results of total phenolic content (TPC), total flavonoid content (TFC), and antioxidant activity of *A. bilimbi* and *D. linearis* extracts from literature review.

Biosource	Extract	TPC (mg GAE/g)	TFC (mg QE/g)	DPPH (%I)	IC_50_ (µg/mL)	Refs.
*D. linearis* leaves	Crude CHCl_3_	0.148 ± 0.002		15.2 ± 0.0 (200 µg/mL)		[12]
	Crude aqueous	31.12 ± 0.06		61.4 ± 2.1 (100 µg/mL)		[6]
	Crude CHCl_3_	10.12 ± 0.05		22.6 ± 0.7 (100 µg/mL)		[11]
	Crude MeOH	34.17 ± 0.05		85.2 ± 0.6 (100 µg/mL)		[11]
*A. bilimbi* leaves	Crude MeOH				10.53 ± 0.72	[21]
	*n*-Hexane				>1000	[21]
	CHCl_3_				13.44 ± 1.00	[21]
	*n*-Butanol				4.14 ± 0.21	[21]
*A. bilimbi* fruits	Crude EtOH		0.851 ± 0.0025			[18]
*A. bilimbi* fruits	Crude MeOH				79.09	[14]
*A. bilimbi* leaves	EtOAc				91.41	[14]
	Crude MeOH				34.85	[14]
*A. bilimbi* leaves	Crude EtOH	53.55 ± 5.11	8.88 ± 1.14			[23]
	Crude aqueous	35.68 ± 4.87	29.71 ± 4.66			[23]

## Data Availability

Data are available upon request.

## Supplementary Materials

The following supporting information can be downloaded at: https://www.mdpi.com/article/10.3390/antiox13111319/s1, Scheme S1: Isolation procedure of **D1**–**D10** from *D. linearis*; Scheme S2: Isolation procedure of **A1**–**A12** from *A. bilimbi*; Table S1: 1H-NMR data (500 MHz) of **D3** and astragalin in DMSO-*d*6; Table S2: NMR data of **D4** and afzelin; Table S3: NMR data of **D5** and isoquercetin; Table S4: NMR data of **D6** and quercetin; Table S5: NMR data of **D8** and kaempferol 3-*O*-*β*-D-glucopyranoside-7-*O*-*α*-L-rhamnopyranoside in methanol-*d*4; Table S6: NMR data of **D9** and 4-vinyl-phenol-1-*O*-[*α*-L-rhamno(1→2)-*β*-D-glucopyranoside; Table S7: NMR data of **D10** and 4-vinyl-phenol-1-*O*-[*α*-L-rhamno(1→6)-*β*-D-glucopyranoside in methanol-*d*4; Table S8: NMR data of **A2** and caffeine in CDCl3; Table S9: NMR data of **A3** and scopoletin in acetone-*d*6; Table S10: 1H-NMR data of **A7** and methyl ferulate in CDCl3; Table S11: 1H-NMR data (500 MHz) of **A9** and cinnamic acid in CDCl3; Table S12: 1H-NMR data (500 MHz) of **A10** and ayanin in acetone-*d*6; Table S13: 1H-NMR data (500 MHz) of **A12** and nobiletin; Table S14: Comparison of NMR data of **A1** and related analogues; Table S15: NMR data of **A1** and **A2** in CDCl3; Figure S1: Isolated compounds from *A. bilimbi*; Figure S2: (A) 1H-NMR spectrum of **A1** in acetone-*d*6; (B) 13C-NMR spectrum of **A1** in acetone-*d*6; (C) HMBC spectrum of **A1** in acetone-*d*6; (D) 1H-NMR spectrum of **A1** in chloroform-*d*; (E) 13C-NMR spectrum of **A1** in chloroform-*d*; (F) HMQC spectrum of **A1** in chloroform-*d*; (G) spectrum of **A1** in chloroform-*d*; Figure S3: (A) HRESI mass spectrum of **D4**; (B) HRESI mass spectrum of **D8**; (C) HRESI mass spectrum of **D9**; (D) HRESI mass spectrum of **D10**; Figure S4: HPLC chromatograms of *A. bilimbi* extracts (AB-crude, AB-H, AB-HEA, AB-EA, and the new compound **A1**; Figure S5: HPLC chromatograms of *D. linearis* extracts (DL-crude, DL-HEA, DL-EA, and DL-water soluble); Figure S6. (A) HPLC chromatogram of **D3**; (B) HPLC chromatogram of **D8**; (C) HPLC chromatogram of **D10**; (D) HPLC chromatogram of **A3**; (E) HPLC chromatogram of **A6**.
