# Supplementary material for: Potential Antioxidant Compounds from the Spores of Dicranopteris linearis and the Branches of Averrhoa bilimbi
Source: Antioxidants (Basel). 2024 Oct 29;13(11):1319. doi: 10.3390/antiox13111319 (PMC11591059; doi:10.3390/antiox13111319)
Supplement: Supplementary file 1 [file antioxidants-13-01319-s001.zip › antioxidants-3210719-supplementary.pdf]

Supplementary Materials

Potential antioxidant compounds from the spores of *Dicranopteris linearis* and the branches of *Averrhoa bilimbi*

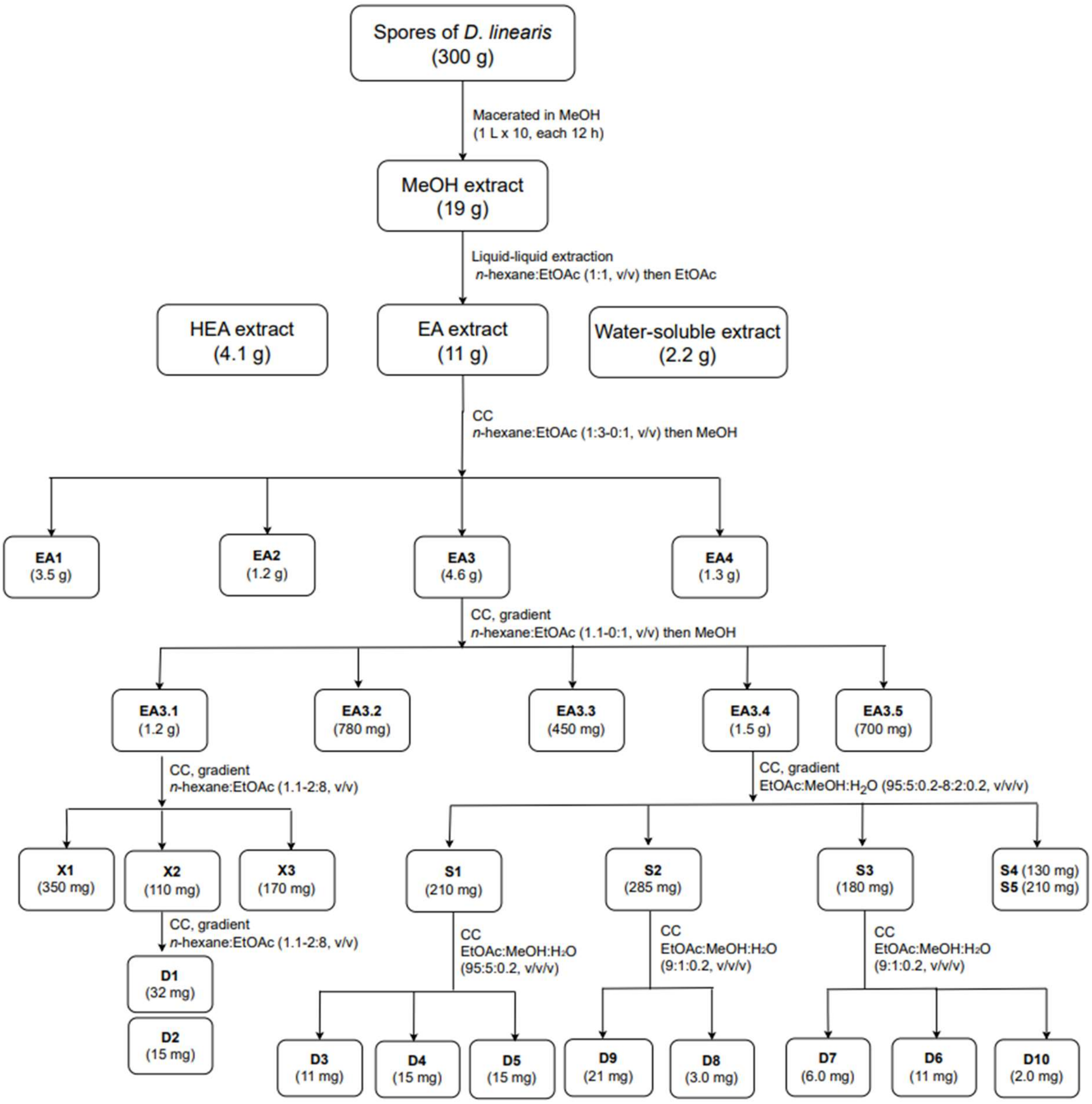

Scheme S1. Isolation procedure of **D1**-**D10** from *D. linearis*

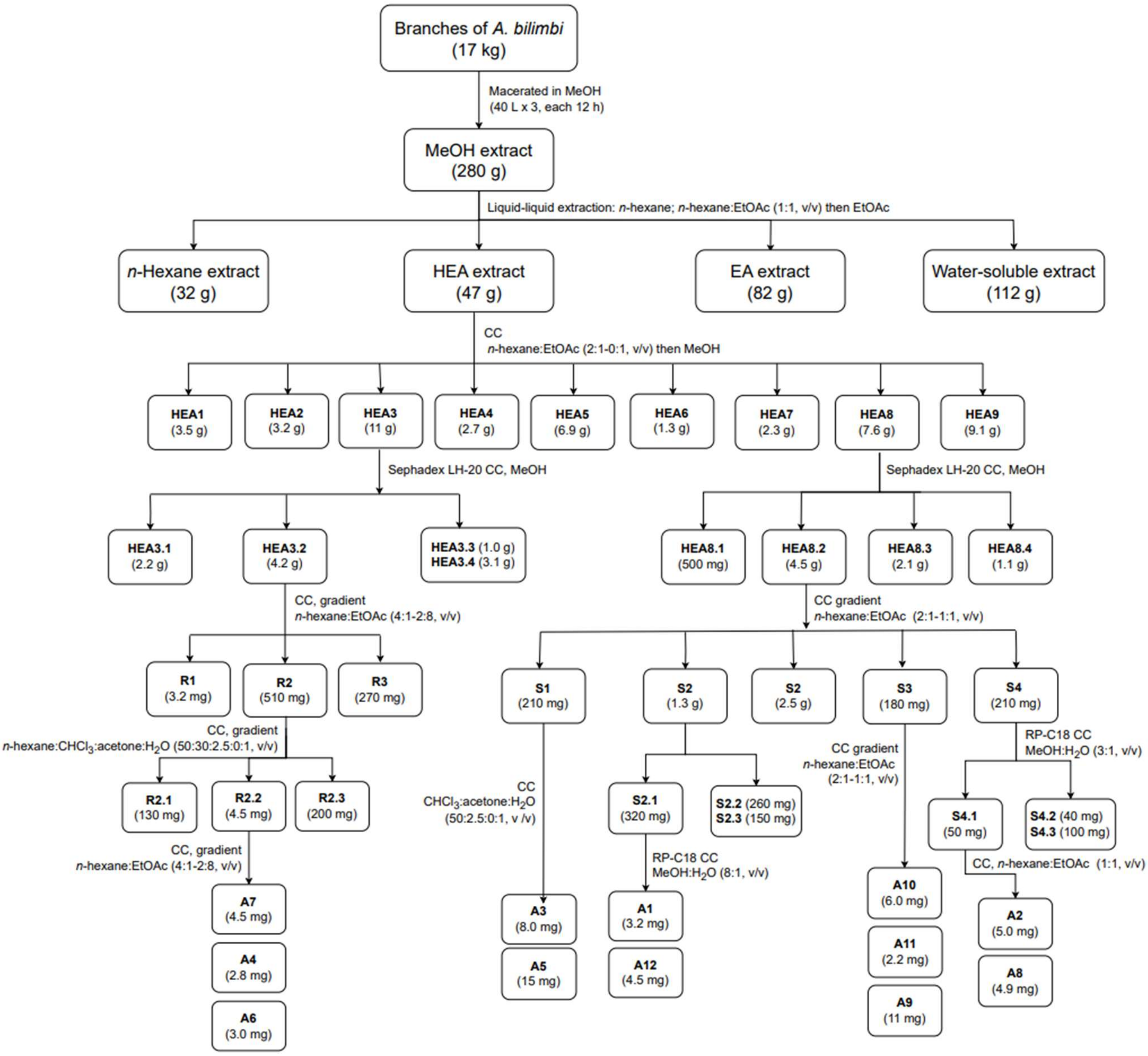

Scheme S2. Isolation procedure of **A1**-**A12** from *A. bilimbi*

Table S1. ^{1}H-NMR data (500 MHz) of **D3** and **astragalin** in DMSO-d_6

No.	D3	Astragalin
	δ_H	δ_H
6	6.20, *d*, *J* = 2.0 Hz	6.20, *d*, *J* = 2.0 Hz
8	6.42, *d*, *J* = 2.0 Hz	6.42, *d*, *J* = 2.0 Hz
5-OH	12.60, *brs*	12.60, *brs*
2′	8.04, *d*, *J* = 9.0 Hz	8.04, *d*, *J* = 9.0 Hz
3′	6.88, *d*, *J* = 9.0 Hz	6.88, *d*, *J* = 9.0 Hz
5′	6.88, *d*, *J* = 9.0 Hz	6.88, *d*, *J* = 9.0 Hz
6′	8.04, *d*, *J* = 9.0 Hz	8.04, *d*, *J* = 9.0 Hz
1′′	5.45, *d*, *J* = 7.5 Hz	5.45, *d*, *J* = 7. Hz 5
2′′	3.20-3.40	3.20-3.40
3′′	3.20-3.40	3.20-3.40
4′′	3.20-3.40	3.20-3.40
5′′	3.20-3.40	3.20-3.40
6′′	3.57, *m*<br>3.19, *m*	3.57, *dd*, *J* = 11.5, 5.5 Hz<br>3.19, *dd*, *J* = 11.5, 5.5 Hz

Ha, K.-N.; Nguyen, T.-V.-A.; Mai, D.-T.; Tran, N.-M.-A.; Nguyen, N.-H.; Vo, G.V.; Duong, T.-H.; Truong Nguyen, H. Alpha-Glucosidase Inhibitors from *Nervilia concolor, Tecoma stans*, and *Bouea macrophylla*. *Saudi Journal of Biological Sciences* **2021**, S1319562X21008731.

Table S2. NMR data of **D4** and **afzelin**

No.	D4		Afzelin	
	δ_H (DMSO-d_6, 500 MHz)	δ_C (DMSO-d_6, 125 MHz)	δ_H (Methanol-d_4, 500 MHz)	δ_C (Methanol-d_4, 125 MHz)
2	-	160.0	-	159.3
3	-	134.2	-	135.8
4	-	177.7	-	179.5
5	-	164.2	-	163.1
6	6.21 (1H, *brs)*	98.7	6.21 (1H, *s*)	99.7
7	-	161.3	-	166.2
8	6.41 (1H, *s*)	93.7	6.38 (1H, *s*)	94.8
9	-	157.2	-	158.7
10	-	104.1	-	105.7
1′	-	121.9	-	122.8
2′	7.76 (1H, *d*, *J* = 8.5 Hz)	120.0	7.32 (1H, *d*, *J* = 8.5 Hz)	117.0
3′	6.92 (1H, *d*, *J* = 8.5 Hz)	148.3	6.92 (1H, *d*, *J* = 8.5 Hz)	146.2
4′	-	144.7		149.8
5′	6.92 (1H, *d*, *J* = 8.5 Hz)	116.0	6.92 (1H, *d*, *J* = 8.5 Hz)	116.4
6′	7.76 (1H, *d*, *J* = 8.5 Hz)	120.9	7.32 (1H, *d*, *J* = 8.5 Hz)	123.0
5-OH	12.64 (1H, *s*)	-	12.64 (1H, *s*)	-
7-OH	10.87 (1H, *s*)	-	10.86 (1H, *s*)	-
3′-OH	9.74 (1H, *s*)	-	9.73 (1H, *s*)	-
4′-OH	9.19 (1H, *s*)	-	9.22 (1H, *s*)	-
1″	5.30 (1H, *s*)	104.1	5.37 (*s*)	103.7
2″	3.09-3.15 (1H, *m*)	70.6	4,23 (1H, d, *J* = 1.5 Hz)	72.0
3″	3.09-3.15 (1H, *m*)	70.3	3.77 (1H, dd, *J* = 9.5, 3.0 Hz)	72.2
4″	3.09-3.15 (1H, *m*)	71.1	3.45-3.42 (*m*)	73.3
5″	3.09-3.15 (1H, *m*)	70.0	3.33-3.22 (*m*)	71.8
6″	0.80 (1H, *d*, *J* = 6.0 Hz)	17.4	0.95 (1H, *d*, *J* = 6.5 Hz)	17.6

Ngoc Mai, T.T.; Minh, P.N.; Phat, N.T.; Duong, T.H.; Minh An, T.N.; Dang, V.S.; Van Hue, N.; Tri, M.D. Antimicrobial and Alpha-Glucosidase Inhibitory Flavonoid Glycosides from the Plant *Mussaenda recurvata* : *In Vitro* and *in Silico* Approaches. *RSC Adv.* **2024**, *14*, 9326–9338.

Table S3. NMR data of **D5** and **isoquercetin**

No.	D5		Isoquercetin	
	δ_H (DMSO-d_6, 500 MHz)	δ_C (DMSO-d_6, 125 MHz)	δ_H (DMSO-d_6, 600 MHz)	δ_C (Methanol-d_4, 100 MHz)
2	-	156.1	-	158.5
3	-	133.3	-	135.6
4	-	177.3	-	179.5
5	-	164.0	-	163.1
6	6.20 (1H, *s*)	98.5	6.20 (1H, *d*, *J* = 2.0 Hz)	99.9
7		161.1		166.0
8	6.40 (1H, *s*)	93.3	6.40 (1H, *d*, *J* = 2.0 Hz)	94.7
9	-	156.1	-	159.0
10	-	103.8	-	105.7
1′	-	121.9	-	123.2
2′	7.52 (1H, *s*)	115.0	7.57 (1H, *dd*, *J* = 2.1, 0.3 Hz)	116.0
3′	-	148.3	-	149.8
4′	-	144.7	-	145.9
5′	6.81 (1H, *d*, *J* = 8.5 Hz)	116.0	6.84 (1H, *dd*, *J* = 8.4, 0.3 Hz)	117.5
6′	7.66 (1H, *d*, *J* = 8.5 Hz)	120.9	7.57 (1H, *dd*, *J* = 8.4, 2.1 Hz)	123.1
5-OH	12.64 (1H, *s*)	-	12.64 (1H, *s*)	-
7-OH	10.87 (1H, *s*)	-	10.86 (1H, *s*)	-
3′-OH	9.74 (1H, *s*)	-	9.73 (1H, *s*)	-
4′-OH	9.19 (1H, *s*)	-	9.22 (1H, *s*)	-
1″	5.37 (1H, *d*, *J* = 8.0 Hz)	101.6	5.46 (1H, *d*, *J* = 7.7 Hz)	104.3
2″	3.24 (1H, *m*)	71.0	3.24 (1H, *ddd*, *J* = 9.1, 7.7, 4.8 Hz)	75.7
3″	3.23 (1H, *m*)	73.0	3.21 (1H, *ddd*, *J* = 9.1, 8.2, 4.8 Hz)	78.1
4″	3.08 (1H, *m*)	67.8	3.08 (1H, *ddd*, *J* = 10.0, 8.2, 5.5 Hz)	71.2
5″	3.07 (1H, *m*)	75.7	3.08 (1H, *ddd*, *J* = 10.0, 5.8, 2.1 Hz)	78.4
6″	3.56 (2H, *m*)	60.0	3.58 (2H, *ddd*, *J* = 11.7, 5.5, 2.1 Hz)	62.6
2″-OH	5.29 (1H, *brs*)	-	5.29 (1H, *d*, *J* = 4.8 Hz)	-
3″-OH	5.14 (1H, *brs*)	-	5.08 (1H, *d*, *J* = 4.8 Hz)	-
4″-OH	4.87 (1H, *brs*)	-	4.96 (1H, *d*, *J* = 5.5 Hz)	-
6″-OH	4.44 (1H, *brs*)	-	4.27 (1H, *dd*/*t*, *J* = 5.8, 5.5 Hz)	-

Zhang, Y.; Wang, D.; Yang, L.; Zhou, D.; Zhang, J. Purification and Characterization of Flavonoids from the Leaves of *Zanthoxylum bungeanum* and Correlation between Their Structure and Antioxidant Activity. *PLoS ONE* **2014**, *9*, e105725.

Table S4. NMR data of **D6** and **quercetin**

No.	D6		Quercitrin	
	δ_H (DMSO-d_6, 500 MHz)	δ_C (DMSO-d_6, 125 MHz)	δ_H (DMSO-d_6, 500 MHz)	δ_C (Methanol-d_4, 125 MHz)
2	-	159.4	-	159.3
3	-	136.0	-	136.2
4	-	179.6	-	179.6
5	-	163.1	-	163.2
6	6.38 (1H, *d*, *J* = 2.0 Hz)	99.9	6.19 (1H, *d*, *J* = 2.1 Hz)	99.8
7	-	165.8	-	165.8
8	6.21 (1H, *d*, *J* = 2.0 Hz)	94.8	6.29 (1H, *d*, *J* = 2.1 Hz)	94.7
9	-	158.5	-	158.5
10	-	105.9	-	105.9
1′	-	122.8	-	123.0
2′	7.52 (1H, *s*)	116.4	7.76 (1H, *d*, *J* = 2.1 Hz)	116.9
3′	-	146.3	-	146.4
4′	-	149.7	-	149.8
5′	6.92 (1H, *d*, *J* = 8.5 Hz)	116.9	6.94 (1H, *d*, *J* = 8.2 Hz)	116.4
6′	7.66 (1H, *d*, *J* = 8.5 Hz)	122.9	7.60 (1H, *dd*, *J* = 8.2, 2.1 Hz)	122.9
5-OH	12.64 (1H, *s*)	-	12.64 (1H, *s*)	-
7-OH	10.87 (1H, *s*)	-	10.86 (1H, *s*)	-
3′-OH	9.74 (1H, *s*)	-	9.73 (1H, *s*)	-
4′-OH	9.19 (1H, *s*)	-	9.22 (1H, *s*)	-
1″	5.35 (1H, *s*)	103.5	5.34 (1H, *d*, *J* = 1.5 Hz)	103.5
2″	4.21 (1H, *m*)	71.9	4.22 (1H, *dd*, *J* = 3.4, 1.8 Hz)	71.9
3″	3.23 (1H, *m*)	72.0	3.75 (1H, *dd*, *J* = 9.5, 3.4 Hz)	72.0
4″	3.08 (1H, *m*)	73.2	3.35 (1H, *d*, *J* = 9.5 Hz)	73.2
5″	3.07 (1H, *m*)	73.0	3.38-3.44 (1H, *m*)	72.1
6″	0.95 (1H, *d*, *J* = 6.0 Hz)	17.6	0.94 (1H, *d*, *J* = 6.4 Hz)	17.6

Napolitano, J.G.; Lankin, D.C.; Chen, S.; Pauli, G.F. Complete ^{1}H NMR Spectral Analysis of Ten Chemical Markers of *Ginkgo biloba*. *Magnetic Resonance in Chemistry* **2012**, *50*, 569–575.

Table S5. NMR data of **D8** and **kaempferol 3-*O*-*β*-D-glucopyranoside-7-*O*-*α*-L-rhamnopyranoside** in methanol-d_4

No.	D8		Kaempferol 3-*O*-*β*-D-glucopyranoside-7-*O*-*α*-L-rhamnopyranoside	
	δ_H (500 MHz)	δ_C (125 MHz)	δ_H (300 MHz)	δ_C (75 MHz)
2	-	159.5	-	159.4
3	-	136.5	-	135.5
4	-	179.6	-	179.6
5	-	-	-	163.2
6	6.23 (*d*, 2.0)	100.0	6.20 (*d*, 1.9)	100.0
7	-	166.0	-	166.0
8	6.41 (*d*, 2.0)	94.9	6.37 (*d*, 1.9)	94.9
9	-	158.6	-	158.6
10	-	106.0	-	106.0
1′	-	122.6	-	122.6
2′	7.78 (*d*, 8.5)	132.0	7.76 (*d*, 8.9)	132.0
3′	6.96 (*d*, 9.0)	116.6	6.94 (*d*, 8.9)	116.6
4′	-	161.7	-	161.7
5′	6.96 (*d*, 9.0)	116.6	6.94 (*d*, 8.9)	116.6
6′	7.78 (*d*, 8.5)	132.0	7.76 (*d*, 8.9)	132.0
1′′	5.72 (*d*, 1.0)	102.6	5.73 (*d*, 1.1)	102.6
2′′	4.29 (*dd*, 3.5, 1.5)	82.6	4.29 (*dd*, 3.5, 1.3)	82.7
3′′	3.83 (*dd*, 10.5, 3.5)	71.8	3.81 (*dd*, 9.3, 3.5)	71.8
4′′	5.14 (*t*, 9.0)	73.4	5.14 (*t*, 9.0)	73.5
5′′	-	72.0	-	72.0
6′′	3.70 (*m*)	17.6	3.70 (*m*)	17.7
1′′′	4.42 (*d*, 8.0)	107.1	4.42 (*d*, 7.7)	107.1
2′′′	-	75.3	-	75.4
3′′′	-	77.9	-	77.9
4′′′	-	70.9	-	71.0
5′′′	-	77.9	-	78.0
6′′′	-	62.4	-	62.4

Hasler, A.; Gross, G.-A.; Meier, B.; Sticher, O. Complex Flavonol Glycosides from the Leaves of *Ginkgo biloba*. *Phytochemistry* **1992**, *31*, 1391–1394.

Table S6. NMR data of **D9** and **4-vinyl-phenol-1-*O*-[*α*-L-rhamno(1→2)-*β*-D-glucopyranoside**

**No.**	**D9**		**4-Vinyl-phenol-1-*O*-[*α*-L-rhamno(1→2)-*β*-D-glucopyranoside**	
	δ_H (DMSO-d_6, 500 MHz)	δ_C (DMSO-d_6, 125 MHz)	δ_H (Methanol-d_4, 500 MHz)	δ_C (Methanol-d_4, 125 MHz)
1	-	157.2	-	157.1
2	6.99 (*d*, 8.5)	116.4	7.03 (*d*, 8.8)	116.4
3	7.40 (*d*, 8.5)	127.2	7.37 (*d*, 8.8)	126.9
4	-	131.2	-	132.2
5	7.40 (*d*, 8.5)	127.2	7.37 (*d*, 8.8)	126.9
6	6.99 (*d*, 8.5)	116.4	7.03 (*d*, 8.8)	116.4
7	6.67 (*dd*, 18.0, 11.0)	136.1	6.69 (*dd*, 17.6, 10.6)	136.0
8	5.14 (*d*, 11.5)<br>5.70 (*d*, 18.0)	112.4	5.13 (*dd*, 11.0, 0.9)<br>5.66 (*dd*, 17.6, 0.9)	111.1
1′	5.16 (*d*, 7.0)	100.5	5.04 (*d*, 7.7)	99.0
2′	3.71 (*m*)	76.6	3.67 (*m*)	77.8
3′	3.67 (*m*)	70.7	3.41 (*m*)	70.8
4′	3.77 (*m*)	66.7	4.04 (*m*)	66.7
5′	3.60 (*m*)	70.0	3.61 (*m*)	71.1
6′	3.85 (*d*, 10.5)<br>3.61 (*m*)	61.0	3.76 (*dd*, 12.1, 2.2)<br>3.70 (*m, overlap*)	61.1
1″	4.55 (*brs*)	100.7	5.30 (*d*, 1.0)	101.1
2″	3.61 (*m*)	70.4	3.95 (*dd*, 3.3, 1.8)	70.8
3″	3.61 (*m*)	77.8	3.61 (*m*)	77.8
4″	3.30 (*m*)	72.0	3.40 (*m*)	72.5
5″	3.60 (*m*)	68.4	4.03 (*m*)	68.5
6″	1.11 (*d*, 6.0)	17.9	1.32 (*d*, 6.4)	16.7
4″-OH	4.75 (*d*, 5.5)	-	-	-

Ponnusamy, Y.; Chear, N.J.-Y.; Ramanathan, S.; Lai, C.-S. Polyphenols Rich Fraction of *Dicranopteris linearis* Promotes Fibroblast Cell Migration and Proliferation *in Vitro*. *Journal of Ethnopharmacology* **2015**, *168*, 305–314.

Table S7. NMR data of **D10** and **4-vinyl-phenol-1-*O*-[*α*-L-rhamno(1→6)-*β*-D-glucopyranoside** in methanol-d_4

No.	D10		4-Vinyl-phenol-1-*O*-[*α*-L-rhamno(1→6)-*β*-D-glucopyranoside	
	δ_H (500 MHz)	δ_C (125 MHz)	δ_H (600 MHz)	δ_C (150 MHz)
1	-	158.8	-	158.8
2	7.03 (*d*, 8.5)	117.8	7.04 (*d*, 8.7)	117.8
3	7.37 (*d*, 9.0)	128.3	7.37 (*d*, 8.7	128.3
4	-	133.6	-	133.5
5	7.37 (*d*, 9.0)	128.3	7.37 (*d*, 8.7)	128.3
6	7.03 (*d*, 8.5)	117.8	7.03 (*d*, 8.7)	117.8
7	6.64 (*m, overlap*)	137.5	6.68 (*dd*, 17.6, 11.0)	137.4
8	5.11 (*dd*, 11.0, 1.0)<br>5.66 (*dd*, 17.5, 0.5)	112.5	5.12 (*dd*, 10.9, 0.7)<br>5.66 (*dd*, 17.6, 0.8)	112.5
1′	4.85 (*overlap*)	102.3	4.86 (*d*, 7.6)	102.3
2′	3.45 (*m*)	75.1	3.48-3.43 (*m*)	75.1
3′	3.33 (*m*)	74.9	3.48-3.43 (*m*)	74.9
4′	3.69 (*m*)	72.4	3.56 (*ddd*, 9.5, 6.6, 1.4)	77.0
5′	3.44 (*m*)	71.6	3.39-3.34 (*m*)	71.6
6′	4.03 (*m*)<br>3.60 (*m*)	67.9	4.03 (*dd*, 10.6, 1.3)<br>3.60 (*dd*, 10.7, 6.6)	67.9
1″	4.70 (*d*, 2.0)	102.2	4.71 (*d*, 1.4)	102.2
2″	3.85 (*m*)	72.2	3.84 (*dd*, 3.3, 1.6)	72.2
3″	3.71 (*m*)	72.4	3.71 (*dd*, 9.5, 3.4)	72.4
4″	3.36 (*m*)	74.0	3.39-3.34 (*m*)	74.0
5″	3.66 (*m*)	69.9	3.66 (*dq*, 9.5, 6.2)	69.9
6″	1.20 (*d*, 6.0)	17.9	1.21 (*d*, 6.2)	17.9

Dall′Acqua, S.; Tomè, F.; Vitalini, S.; Agradi, E.; Innocenti, G. *In Vitro* Estrogenic Activity of *Asplenium trichomanes* L. Extracts and Isolated Compounds. *Journal of Ethnopharmacology* **2009**, *122*, 424–429.

Table S8. NMR data of **A2** and **caffeine** in $CDCl_3$

No.	A2		Caffeine	
	δ_H (500 MHz)	δ_C (125 MHz)	δ_H (500 MHz)	δ_C (125 MHz)
1	3.41	27.8	3.37	27.5
2	-	150.7	-	151.3
3	3.59	29.6	3.55	29.3
4	-	149.5	-	148.3
5	-	107.8	-	107.1
6	-	154.7	-	154.9
7	3.99	33.5	4.01	33.2
8	7.51	141.8	7.58	141.2

Octaviana, L.; Hakim, E.H. Two Arylpropanoid Derivatives from the Leaves of *Morus cathayana*. *Proceeding of The International Seminar on Chemistry* **2008**, 262–264.

Table S9. NMR data of **D3** and **scopoletin** in acetone-d_6

No.	A3		Scopoletin	
	δ_H (500 MHz)	δ_C (125 MHz)	δ_H (500 MHz)	δ_C (125 MHz)
1	-	160.8	-	160.8
2	6.18 (1H, *d*, *J* = 9.5 Hz)	115.8	6.25 (1H, *d*, *J* = 9.75 Hz)	113.3
3	7.85 (1H, *d*, *J* = 9.5 Hz)	144.7	7.84 (1H, *d*, *J* = 9.75 Hz)	144.7
4	-	113.4	-	112.1
5	7.20 (1H, *s*)	110.1	7.19 (1H, *s*)	109.9
6	-	146.0	-	146.0
7	-	151.9	-	151.9
8	6.80 (1H, *s*)	103.8	6.79 (1H, *s*)	103.7
9	-	-	-	151.2
10	3.90 (3H, *s*)	56.8	3.90 (3H, *s*)	56.7

Octaviana, L.; Hakim, E.H. Two Arylpropanoid Derivatives from the Leaves of *Morus cathayana*. *Proceeding of The International Seminar on Chemistry* **2008**, 262–264.

Table S10. ^{1}H-NMR data of **A7** and **methyl ferulate** in $CDCl_3$

No.	A7	Methyl ferulate
	δ_H (500 MHz)	δ_H (400 MHz)
2	7.03 (1H, *d*, 1.5)	7.02 (1H, *d*, 2.0)
5	6.93 (1H, *d*, 8.0)	6.91 (1H, *d*, 8.0)
6	7.08 (1H, *dd*, 8.0, 1.5)	7.06 (1H, *dd*, 8.0, 2.0)
7	7.63 (1H, *d*, 16.0)	7.63 (1H, *d*, 16.0)
8	6.30 (1H, *d*, 16.0)	7.28 (1H, *d*, 16.0)
10	3.80 (3H, *s*)	3.79 (3H, *s*)
11	3.93 (3H, *s*)	3.92 (3H, *s*)

Ramadan, A.M.A.; Zidan, S.A.H.; Shehata, R.M.; El-Sheikh, H.H.; Ameen, F.; Stephenson, S.L.; Al-Bedak, O.A.-H.M. Antioxidant, Antibacterial, and Molecular Docking of Methyl Ferulate and Oleic Acid Produced by *Aspergillus pseudodeflectus* AUMC 15761 Utilizing Wheat Bran. *Scientific Reports* **2024**, *14*, 3183–3205.

Table S11. ^{1}H-NMR data (500 MHz) of **A9** and **cinnamic acid** in $CDCl_3$

No.	A9	Cinnamic acid
	δ_H	δ_H
2	6.46 (1H, *d*, *J* = 16.0 Hz)	6.46 (1H, *d*, *J* = 15.5 Hz)
3	7.79 (1H, *d*, *J* = 16.0 Hz)	7.80 (1H, *d*, *J* = 16.0 Hz)
2′	7.54-7.56 (2H, *m*)	7.55-7.57 (2H, *m*)
3′	7.39-7.42 (3H, *m*)	7.39-7.42 (3H, *m*)
4′	7.39-7.42 (3H, *m*)	7.39-7.42 (3H, *m*)
5′	7.39-7.42 (3H, *m*)	7.39-7.42 (3H, *m*)
6′	7.54-7.56 (2H, *m*)	7.55-7.57 (2H, *m*)

Ngoc, D.T.B.; Dieu, N.T.H.; Huy, D.T. Some Flavonoids the Leaves of *Combretum quadrangulare* Growing in Vietnam. *Ho Chi Minh City University of Education Journal of Science* **2021**, *18*, 1359–1367.

Table S12. ^{1}H-NMR data (500 MHz) of **A10** and **ayanin** in acetone-d_6

No.	A10	Ayanin
	δ_H	δ_H
6	6.32 (1H, *d*, *J* = 2.0 Hz)	6.31 (1H, *d*, *J* = 2.1 Hz)
8	6.67 (1H, *d*, *J* = 2.0 Hz)	6.66 (1H, *d*, *J* = 2.3 Hz)
2′	7.72-7.69 (1H, *m*)	7.72-7.70 (1H, *m*)
5′	7.01 (1H, *d*, *J* = 8.5 Hz)	7.01 (1H, *d*, *J* = 8.5 Hz)
6′	7.71 (1H, *dd*, *J* = 8.5, 2.0 Hz)	7.79 (1H, *dd*, *J* = 8.5, 2.1 Hz)
5-OH	12.75 (1H, *s*)	12.75 (1H, *brs*)
3′-OH	8.49 (1H, *s*)	8.49 (1H, *brs*)
3-OMe	3.89 (3H, *s*)	3.90 (1H, *s*)
7-OMe	3.91 (3H, *s*)	3.94 (3H, *s*)
4′-OMe	3.94 (3H, *s*)	3.91 (3H, *s*)

Ngoc, D.T.B.; Dieu, N.T.H.; Huy, D.T. Some Flavonoids the Leaves of *Combretum quadrangulare* Growing in Vietnam. *Ho Chi Minh City University of Education Journal of Science* **2021**, *18*, 1359–1367.

Table S13. ^{1}H-NMR data (500 MHz) of **A12** and **nobiletin**

No.	A12	Nobiletin
	δ_H ($CDCl_3$)	δ_H (Acetone-d_6)
3	6.62 (*s*)	6.63 (*s*)
2′	7.42 (*d*, 2.5)	7.59 (*d*, 1.5)
5′	7.00 (*d*, 8.5)	7.16 (*d*, 8.5)
6′	7.58 (*dd*, 8.5, 2.5)	7.66 (*dd*, 8.0, 1.5)
5-OCH_3	3.95 (*s*)	3.58 (*s*)
6-OCH_3	3.95 (*s*)	3.89 (*s*)
7-OCH_3	4.02 (*s*)	4.00 (*s*)
8-OCH_3	4.10 (*s*)	4.09 (*s*)
3′-OCH_3	3.96 (*s*)	3.97 (*s*)
4′-OCH_3	3.96 (*s*)	3.92 (*s*)

Nguyen, N.; Duong, T.; Truong Nguyen, H.; Vu, Y.T.; Tran, T.; Ho, T.; Mai, C.; Mai, D.; Nguyen, H.; Thuy Le, H.; et al. New Halogenated Flavonoids from *Adenosma bracteosum* and *Vitex Negundo* and Their α-Glucosidase Inhibition. *Chemistry & Biodiversity* **2023**, e202300390.

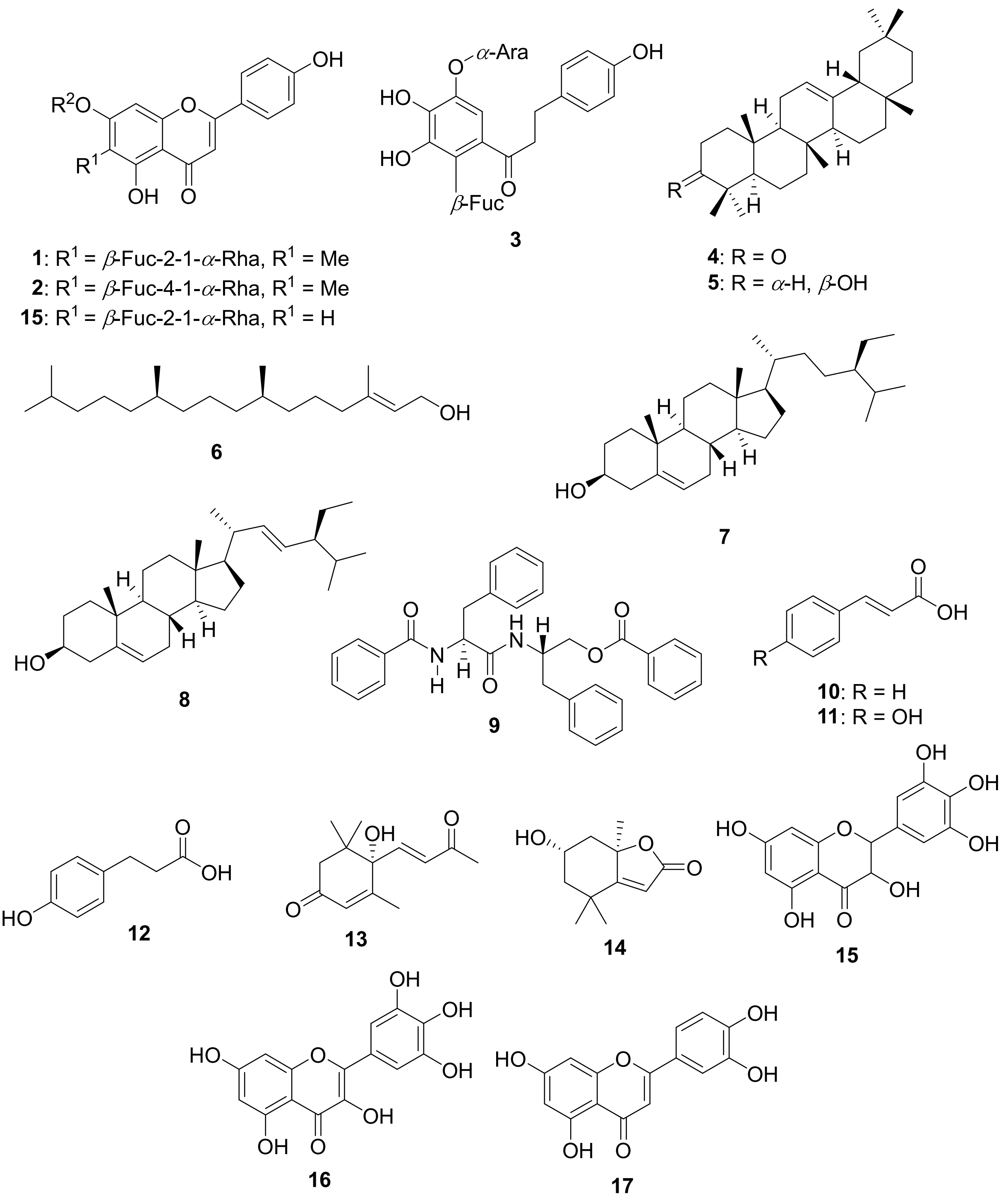

Figure S1. Isolated compounds from *A. bilimbi*

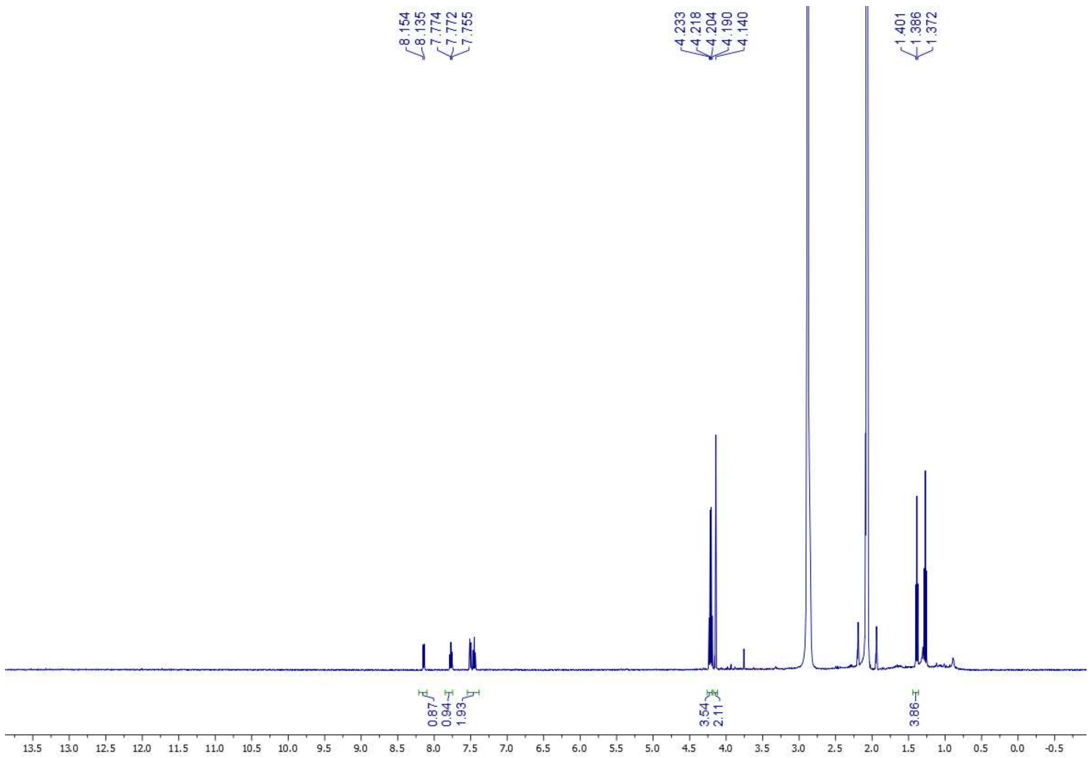

Figure S2A. ^{1}H-NMR spectrum of **A1** in acetone-d_6

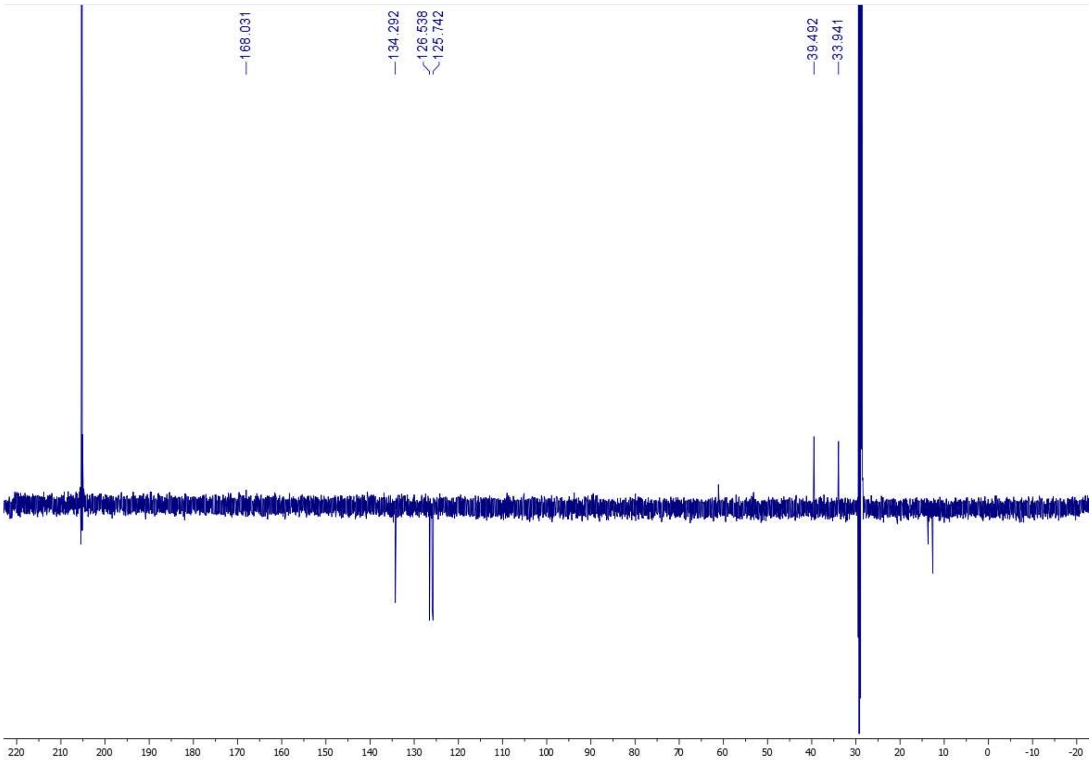

Figure S2B. ^{13}C-NMR spectrum of **A1** in acetone-d_6

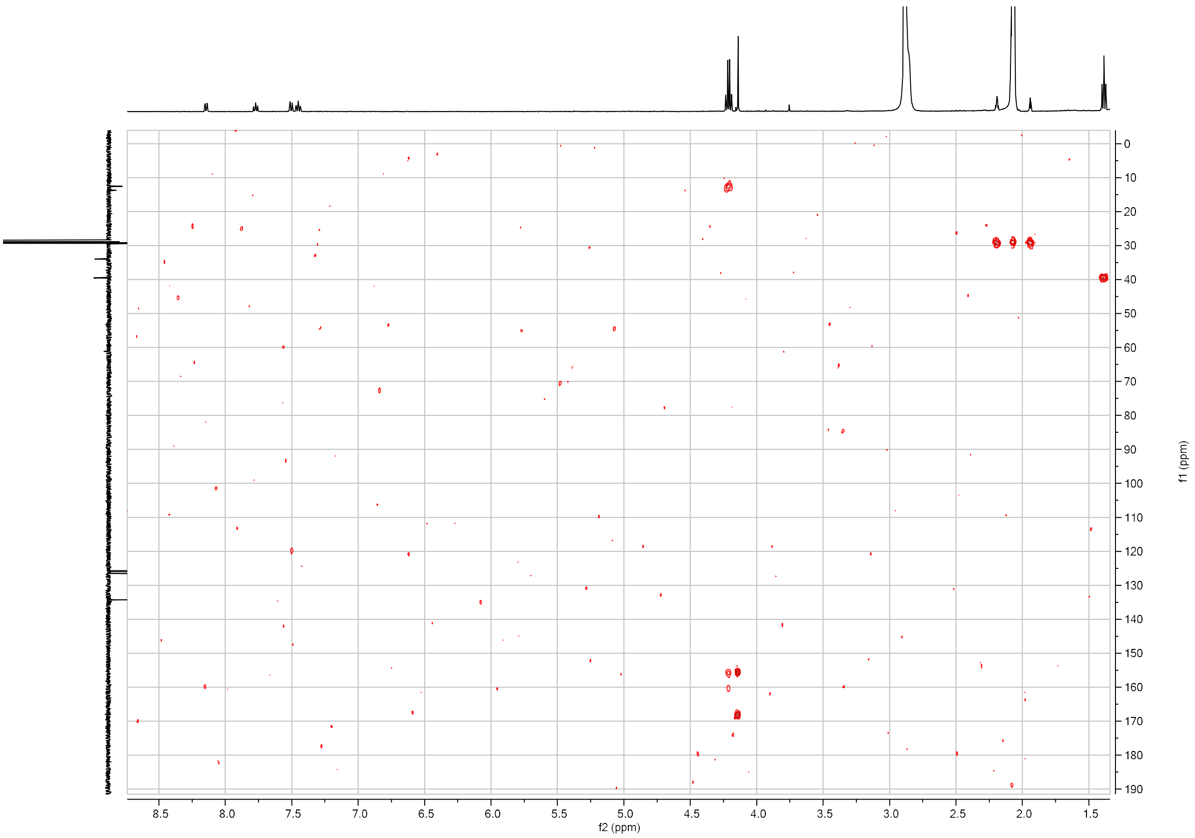

Figure S2C. HMBC spectrum of **A1** in acetone-d_6

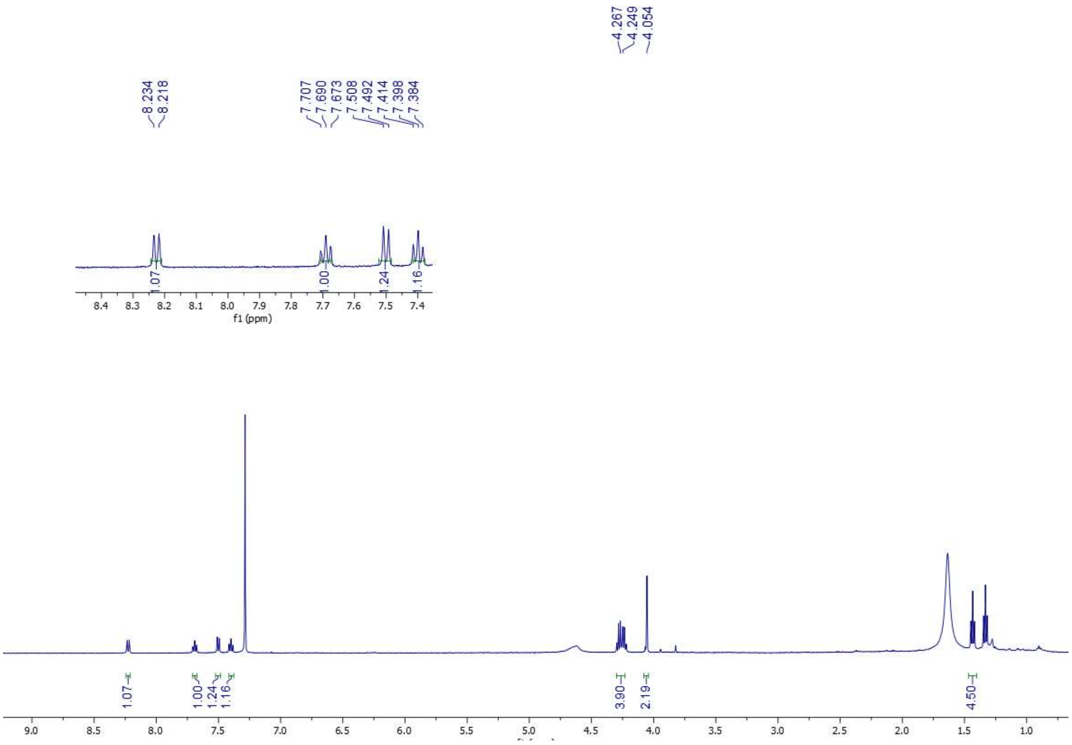

Figure S2D. ^{1}H-NMR spectrum of **A1** in chloroform-d

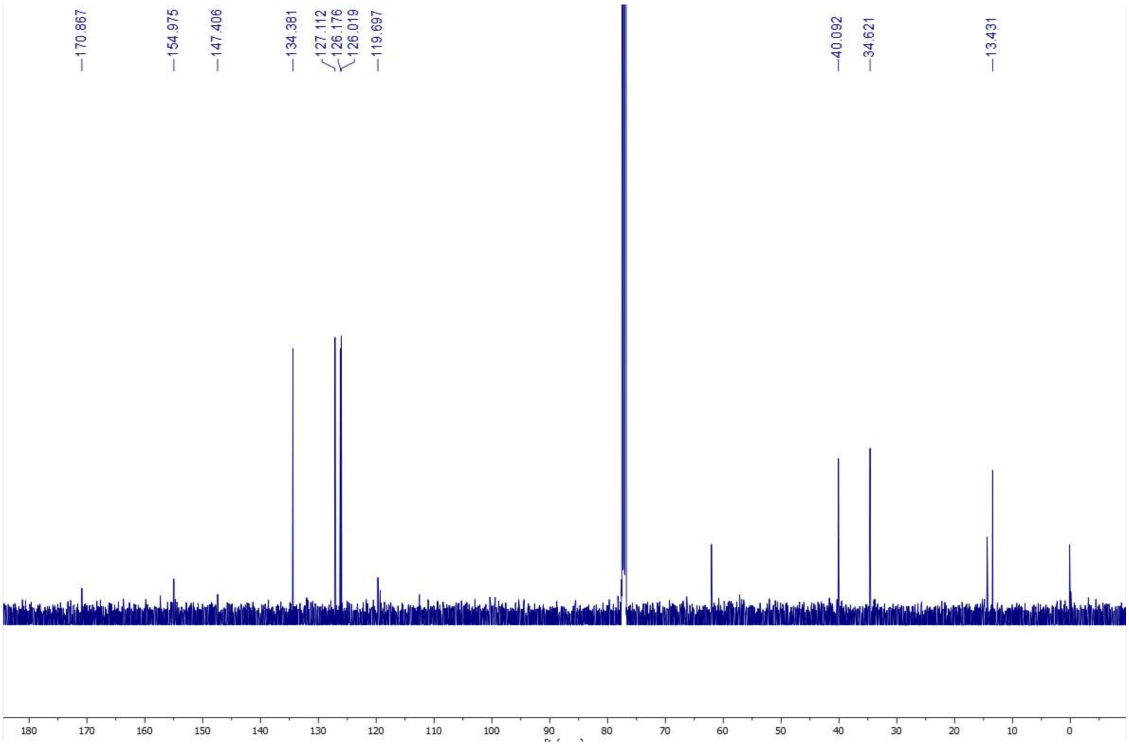

Figure S2E. ^{13}C-NMR spectrum of **A1** in chloroform-*d*

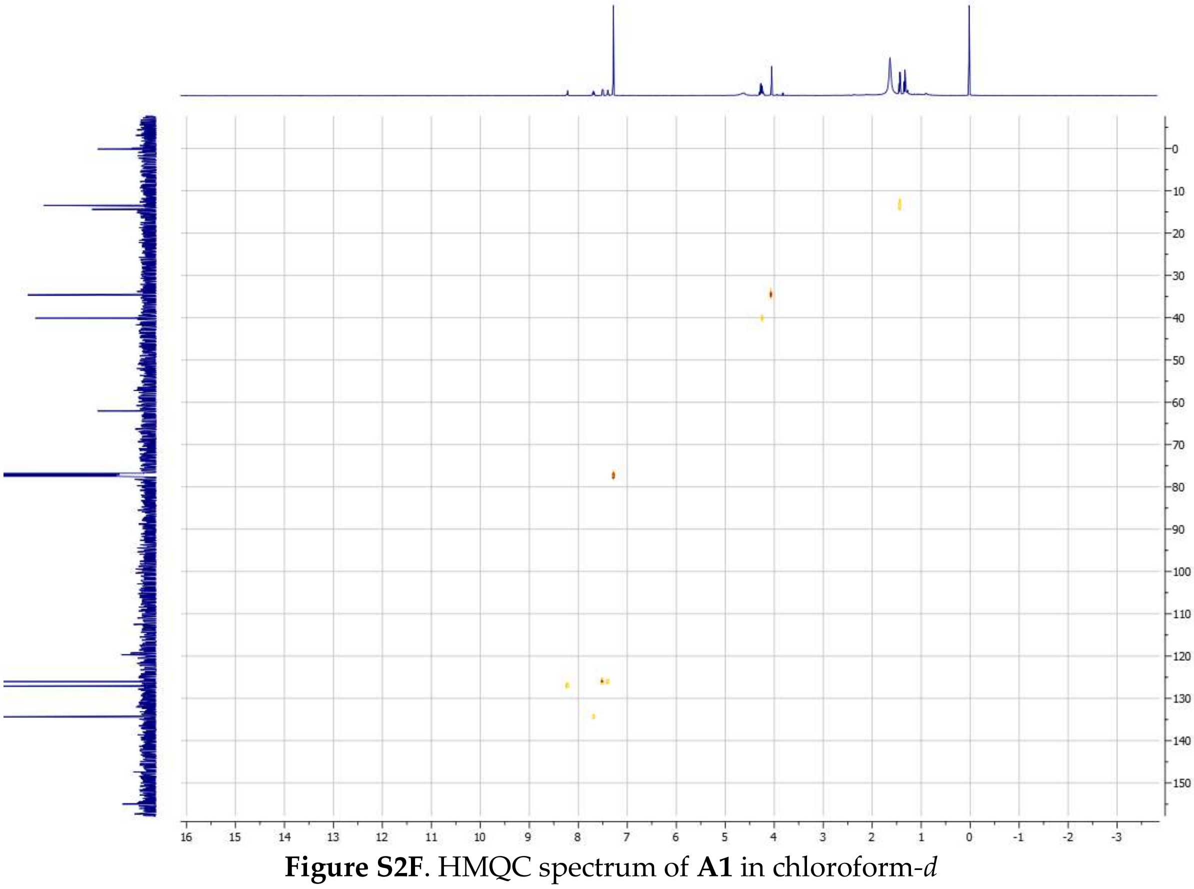

Figure S2F. HMQC spectrum of **A1** in chloroform-*d*

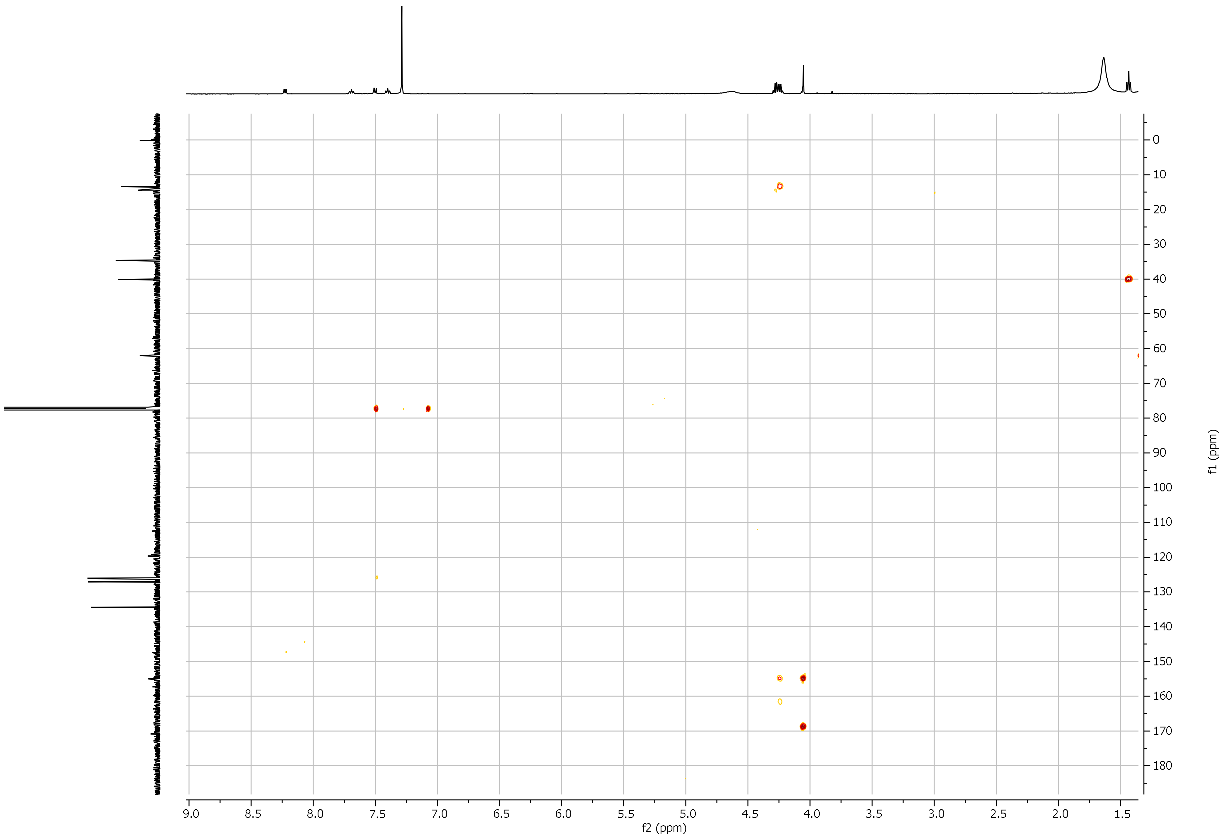

Figure S2G. HMBC spectrum of **A1** in chloroform-*d*

Table S14. Comparison of NMR data of **A1** and related analogues.

No.	**A1**		**2,2′-(2,4-Dioxoquinazoline-1,3(2*H*,4*H*)-*d*iyl)di(acetohydrazide)**		**2,2′-(2,4-Dioxoquinazolin-1,3(2*H*,4*H*)-diyl)bis(N′-(2-chloroacetyl)aceto hydrazide)**		**A2 (Caffeine)**	
	δ_H (Acetone-d_6, 500 MHz)	δ_C (Acetone-d_6, 125 MHz)	δ_H (DMSO-d_6, 400 MHz)	δ_C (DMSO-d_6, 100 MHz)	δ_H (DMSO-d_6, 400 MHz)	δ_C (DMSO-d_6, 100 MHz)	δ_H (CDCl$_3$, 500 MHz)	δ_C (CDCl$_3$, 125 MHz)
1	-	161.3	-	161.0	-	160.9	-	151.4
3	-	156.7		150.5	-	150.8	-	148.7
5	7.49 (1H, *d*, *J* = 8.5 Hz)	126.7	7.24 (1H, *d*, *J* = 8.76 Hz)	115.1		123.3	-	-
6	7.77 (1H, *ddd*, *J* = 8.5, 7.0, 1.5 Hz)	135.2	7.71 (1H, *t*, *J* = 7.01 Hz)	135.3	7.15 - 8.11 (4H, *m*)	135.6	7.71 (1H, *s*)	107.4
7	7.45 (1H, *ddd*, *J* = 8.0, 7.0, 1.0 Hz)	126.9	7.33 (1H, *t*, *J* = 6.98 Hz)	123.0		123.7	-	-
8	8.13 (1H, *dd*, *J* = 8.0, 1.5 Hz)	127.5	8.06 (1H, *d*, *J* = 8.59 Hz)	127.9		128.6	-	155.3
9	-	119.7	-	-	-	114.8	-	141.1
10	-	147.0	-	140.4	-	140.5	-	-
1′	4.21 (2H, *q*, *J* = 7.0 Hz)	40.1	4.74 (2H, *s*)	42.6	4.91 (2H, *s*)	40.1	3.41 (3H, *s*)	27.9
1″	1.38 (3H, *t*, *J* = 7.0 Hz)	14.4	-	165.9	-	164.7	-	-
2′	4.12 (2H, *s*)	34.6	4.52 (2H, *s*)	45.0	4.67 (2H, *s*)	44.5	3.59 (3H, *s*)	29.7
2″	-	169.0	-	169.9	-	166.0	-	-
1′-CH$_2$-Cl	-	-	-	-	4.14 (2H, *s*)	41.2	3.99 (3H, *s*)	33.6
2′-CH$_2$-Cl	-	-	-	-	4.16 (2H, *s*)	42.3	-	-
-NH$_2$	-	-	4.27 (4H, *brs*)	-	-	-	-	-
1‴-NH	-	-	9.30 (1H, *s*)	-	10.49 (4H, *s*)	-	-	-
2‴-NH	-	-	9.21 (1H, *s*)	-		-	-	-

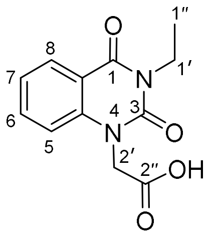

A1

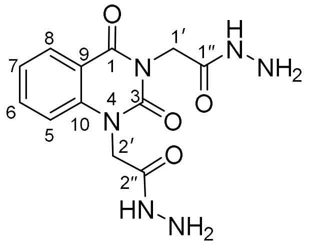

2,2′-(2,4-Dioxoquinazoline-1,3(2*H*,4*H*)-diyl)di(acetohydrazide)

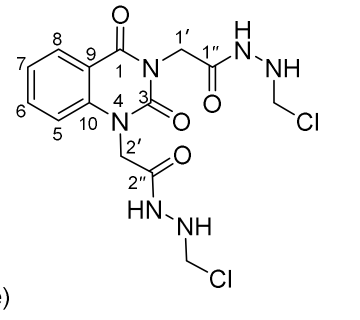

2,2′-(2,4-Dioxoquinazolin-1,3(2*H*,4*H*)-diyl) bis(*N*′-(2-chloroacetyl)acetohydrazide)

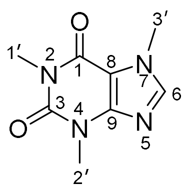

Caffein

Table S15. NMR data of **A1** and **A2** in $CDCl_3$

No.	**A2**		**A1**	
	δ_H (500 MHz)	δ_C (125MHz)	δ_H (500 MHz)	δ_C (125MHz)
1	-	151.4	-	161.0
3	-	148.7	-	154.9
5	-	-	7.51 (1H, *d*, *J* = 8.0 Hz)	126.0
6	7.71 (1H, *s*)	107.4	7.71 (1H, *t*, *J* = 8.5 Hz)	134.4
7	-	-	7.41 (1H, *t*, *J* = 8.0, 7.0 Hz)	126.0
8	-	155.3	8.23 (1H, *d*, *J* = 8.0 Hz)	127.0
9	-	141.1	-	119.7
10	-	-	-	147.0
1′	3.41 (3H, *s*)	27.9	4.27 (2H, *m*)	40.1
1″	-	-	1.31 (3H, *t*, *J* = 7.5, 7.0 Hz)	14.4
2′	3.59 (3H, *s*)	29.7	4.03 (2H, *s*)	34.6
2″	-	-	-	170.9
3′	3.99 (3H, *s*)	33.6	-	-

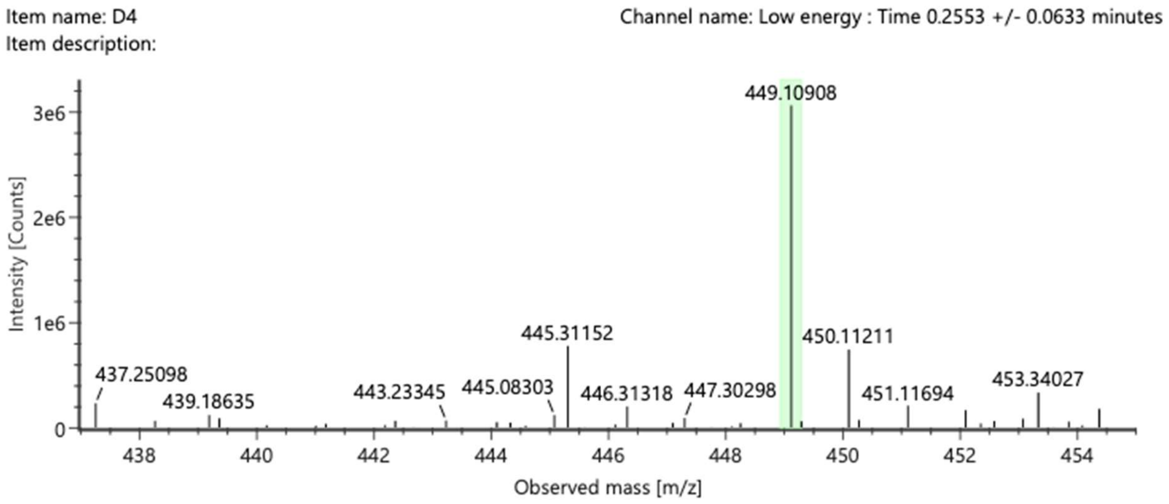

Figure S3A. HRESI mass spectrum of **D4**

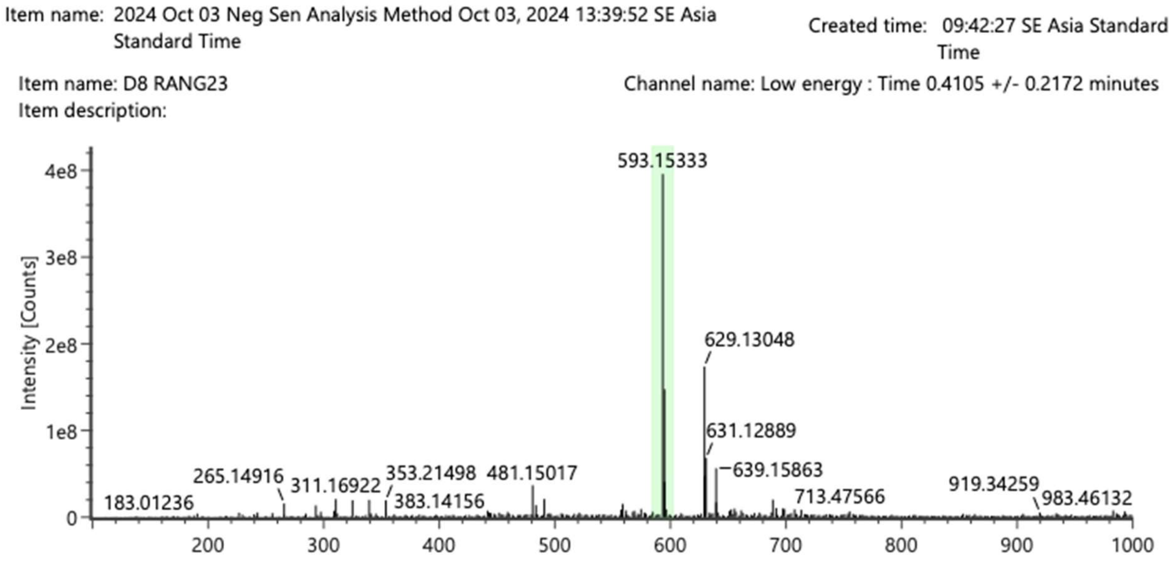

Figure S3B. HRESI mass spectrum of **D8**

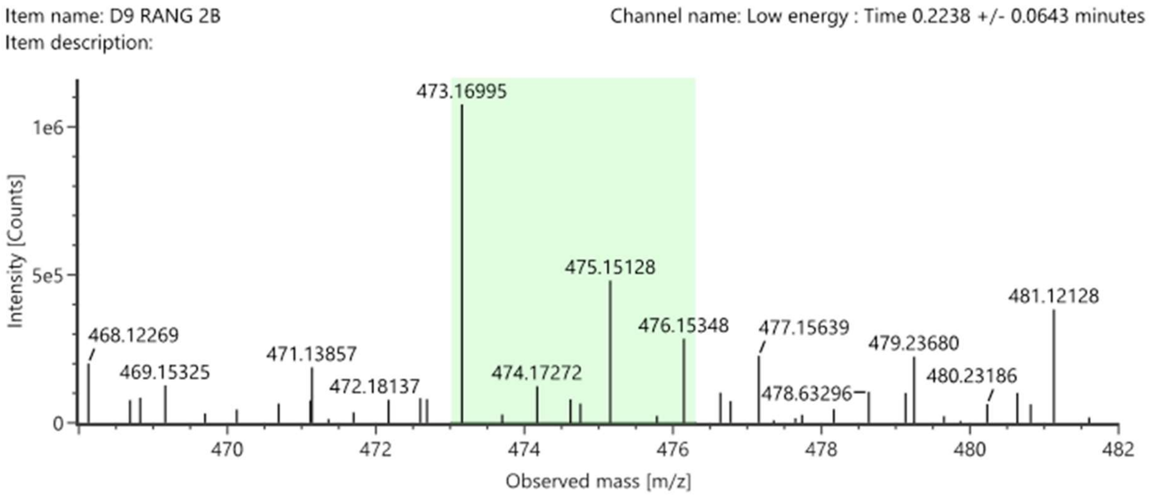

Figure S3C. HRESI mass spectrum of **D9**

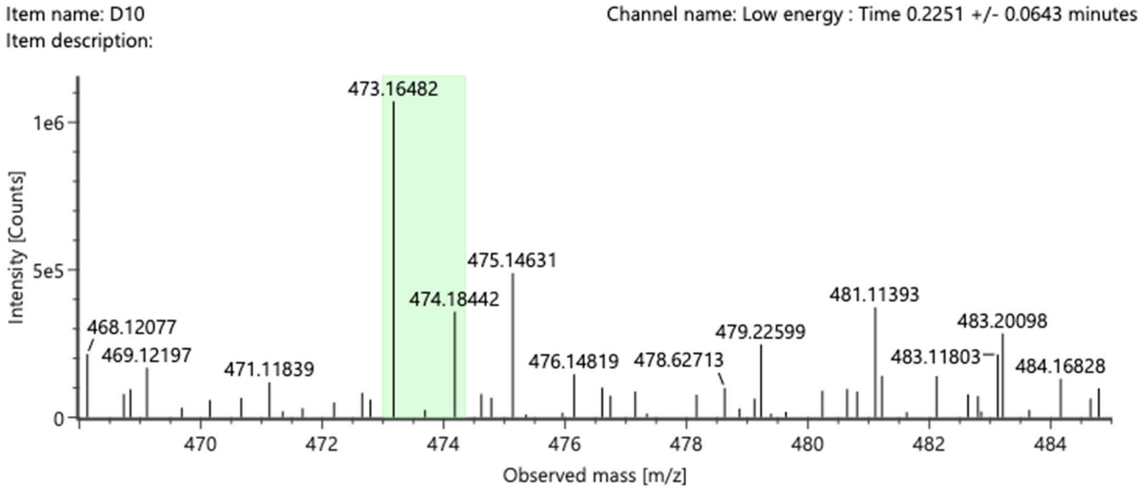

Figure S3D. HRESI mass spectrum of **D10**

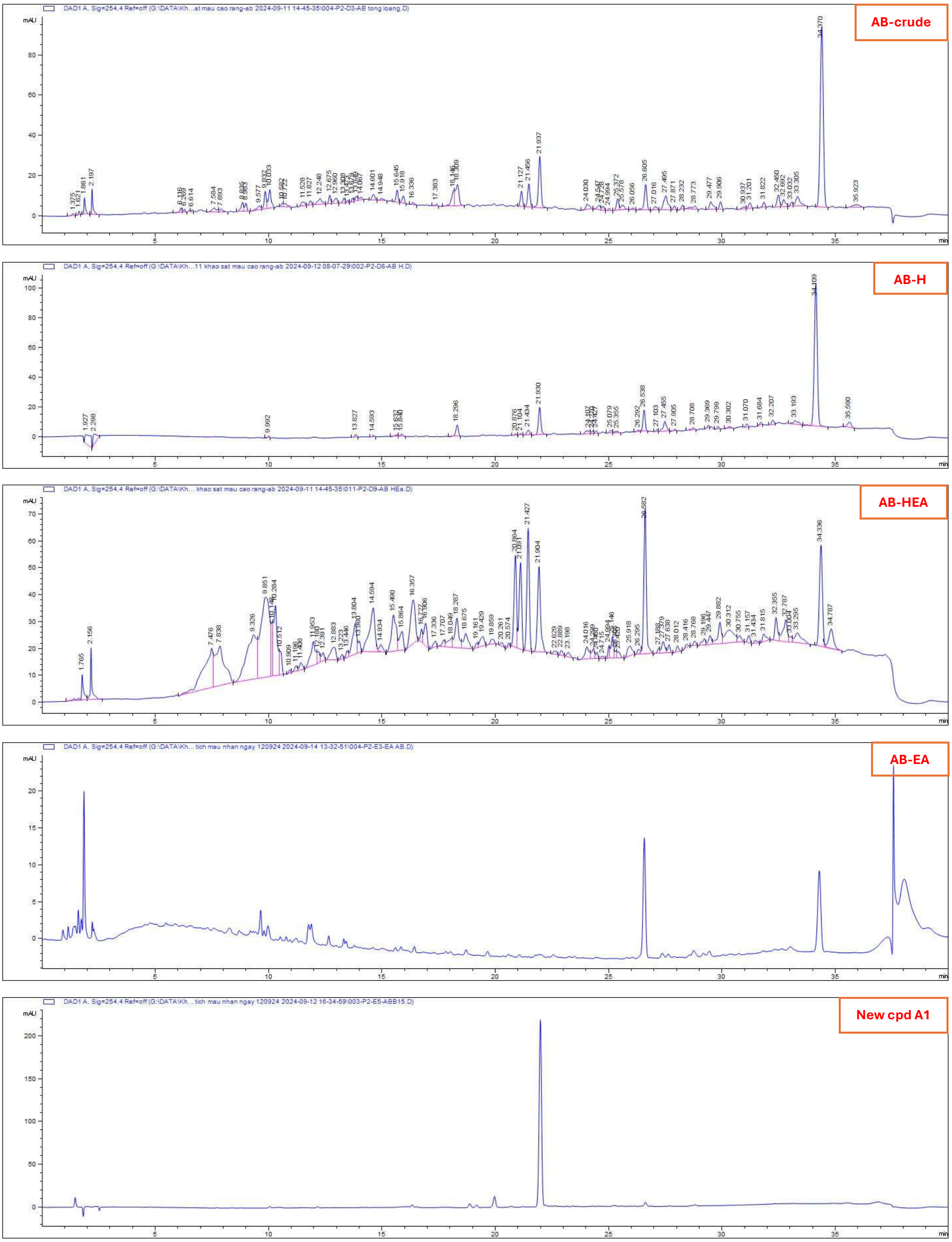

Figure S4. HPLC chromatograms of *A. bilimbi* extracts (AB-crude, AB-H, AB-HEA, AB-EA, and the new compound **A1**).

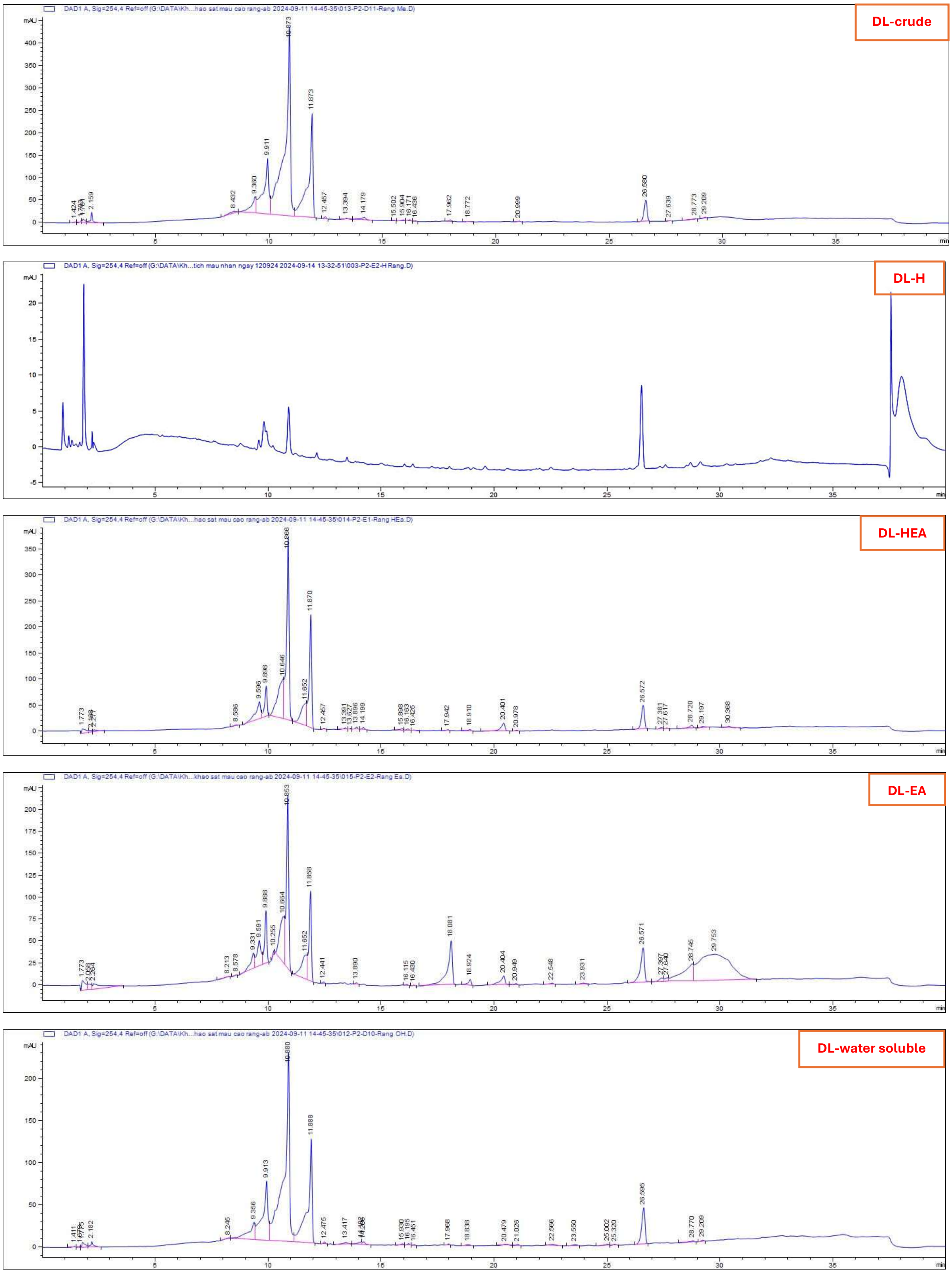

Figure S5. HPLC chromatograms of *D. linearis* extracts (DL-crude, DL-HEA, DL-EA, and DL-water soluble).

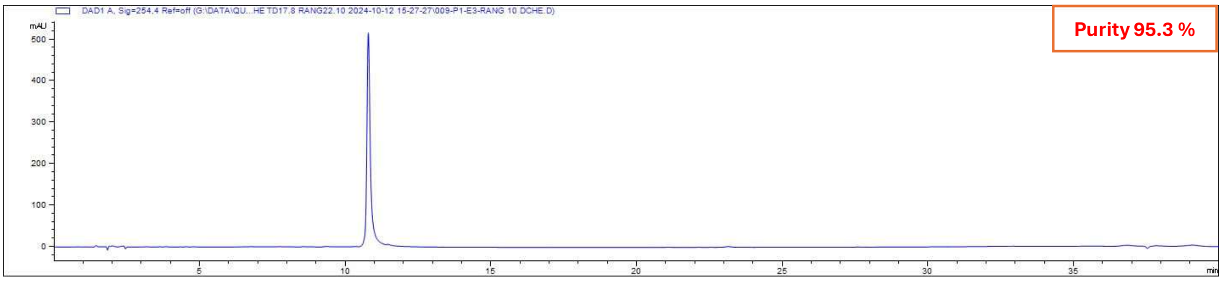

Figure S6A. HPLC chromatogram of **D3**

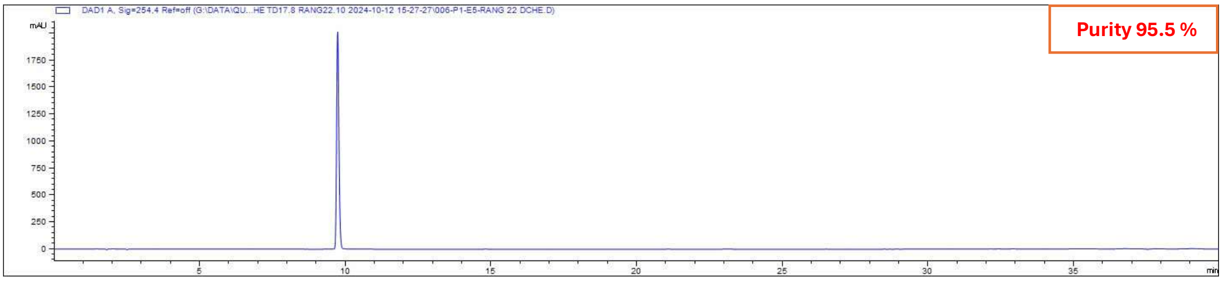

Figure S6B. HPLC chromatogram of **D8**

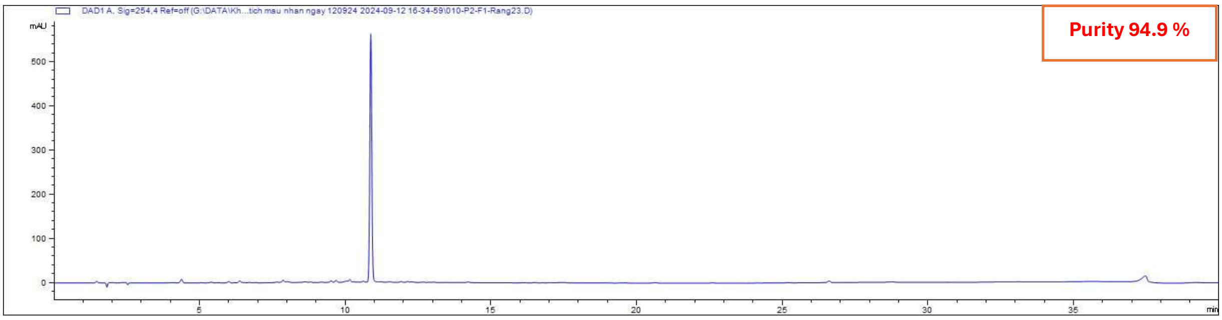

Figure S6C. HPLC chromatogram of **D10**

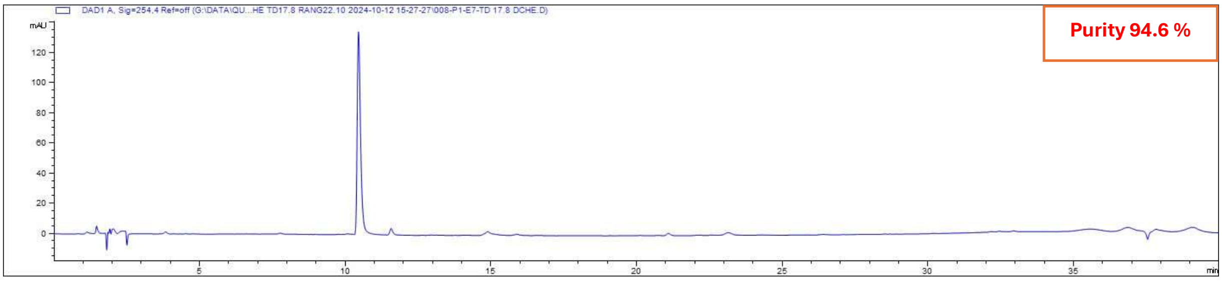

Figure S6D. HPLC chromatogram of **A3**

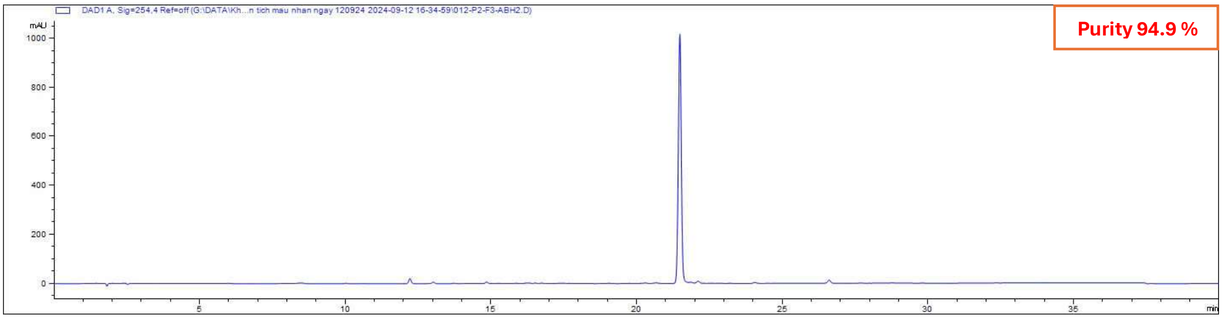

Figure S6E. HPLC chromatogram of **A6.**
